# Targeting CCL2-CCR4 axis suppress cell migration of head and neck squamous cell carcinoma

**DOI:** 10.1038/s41419-022-04610-5

**Published:** 2022-02-17

**Authors:** Zihang Ling, Wei Li, Jiaqi Hu, Yuanyuan Li, Miao Deng, Siyuan Zhang, Xianyue Ren, Tong Wu, Juan Xia, Bin Cheng, Xiaoan Tao

**Affiliations:** 1 Hospital of Stomatology, Guanghua School of Stomatology, Sun Yat-sen University, Guangzhou, Guangdong 510055 P. R. China; 2 Guangdong Provincial Key Laboratory of Stomatology, Guangzhou, Guangdong 510055 P. R. China

**Keywords:** Head and neck cancer, Cell migration, Cancer therapy

## Abstract

For head and neck squamous cell carcinoma (HNSCC), the local invasion and distant metastasis represent the predominant causes of mortality. Targeted inhibition of chemokines and their receptors is an ongoing antitumor strategy established on the crucial roles of chemokines in cancer invasion and metastasis. Herein, we showed that C-C motif chemokine ligand 2 (CCL2)- C-C motif chemokine receptor 4 (CCR4) signaling, but not the CCL2- C-C motif chemokine receptor 2 (CCR2) axis, induces the formation of the vav guanine nucleotide exchange factor 2 (Vav2)- Rac family small GTPase 1 (Rac1) complex to activate the phosphorylation of myosin light chain (MLC), which is involved in the regulation of cell motility and cancer metastasis. We identified that targeting CCR4 could effectively interrupt the activation of HNSCC invasion and metastasis induced by CCL2 without the promoting cancer relapse observed during the subsequent withdrawal period. All current findings suggested that CCL2-CCR4-Vav2-Rac1-p-MLC signaling plays an essential role in cell migration and cancer metastasis of HNSCC, and CCR4 may serve as a new potential molecular target for HNSCC therapy.

## Introduction

Cell migration is a crucial process for the invasive and metastasis of cancer [1, 2]. An increasing number of evidence has revealed that targeted drugs that disrupt cell migration led to the significant improvement in five-year survival rates of prostate and breast cancer patients with metastasis [3]. Notably, chemokines and their receptors are essential coordinators of directed migration of cancer cells and cell–cell interactions and significantly impact tumor development. Therefore, targeted inhibition of chemokines or their receptors represents a persistent focus for optimizing antitumor strategy [4].

CCL2, also known as monocyte chemotactic protein-1 (MCP-1), has been shown to play critical roles in regulating tumor development and progression [5–11]. Although some encouraging results have been achieved in targeting CCL2 or its receptors as an antitumoral strategy, three clinical trials targeting the CCL2-CCR2 axis with the humanized neutralizing anti-CCR2 mAb (MLN1202) and a humanized monoclonal CCL2 neutralizing antibody (CNTO 888) were unsuccessful in suppressing tumor growth and metastasis in solid tumors [12–14]. More importantly, a recent study indicated that directly targeting CCL2 may provoke unexpected adverse effects, indicating that cessation of CCL2 inhibition leads to a rebound in the number of circulating monocytes, increasing angiogenesis, promoting metastases, and accelerating death in the breast cancer model [15]. Therefore, avoiding the adverse effects during the direct intervention of CCL2 represents an essential challenge of current CCL2-CCR2 axis-targeted antitumor therapy.

CCR4, another important receptor of CCL2, is overexpressed in many solid tumors and hematologic malignancies. A humanized anti-CCR4 antibody, Mogamulizumab, has been applied to treat relapsed/refractory adult T-cell leukemia (ATL) and cutaneous T-cell lymphoma (CTL) in Japan [16, 17]. In some solid tumors, an increasing number of studies have shown that CCL2, CCL17, and CCL22 with CCR4 expression induced cancer cells migration, EMT, and metastasis in some solid tumors [18–22]. Given that CCR4 deficiency does not affect the infiltration and migration of monocytes, CCR4 inhibition to block the CCL2/CCR4 axis could be a promising novel antitumoral strategy to reduce the risk of rapid tumor recurrence and metastasis during the cessation of CCL2 inhibition treatment.

Local invasion and distant metastasis remain the primary cause of mortality in patients with HNSCC [23]. In the present study, we demonstrated that CCL2/CCR4 interaction, but not CCL2/CCR2 interaction, promoted HNSCC cell migration and invasion by inducing the formation of Vav2-Rac1 complex to upregulate active Rac1 level. Targeting CCR4 could be a promising migrastatics strategy to reduce cancer cell motility and metastasis in HNSCC without promoting tumor relapse observed during the interruption of CCL2 inhibition.

## Results

### Overexpression of CCL2 was inherent and predicted poor prognosis in HNSCC

**Fig. 1:**
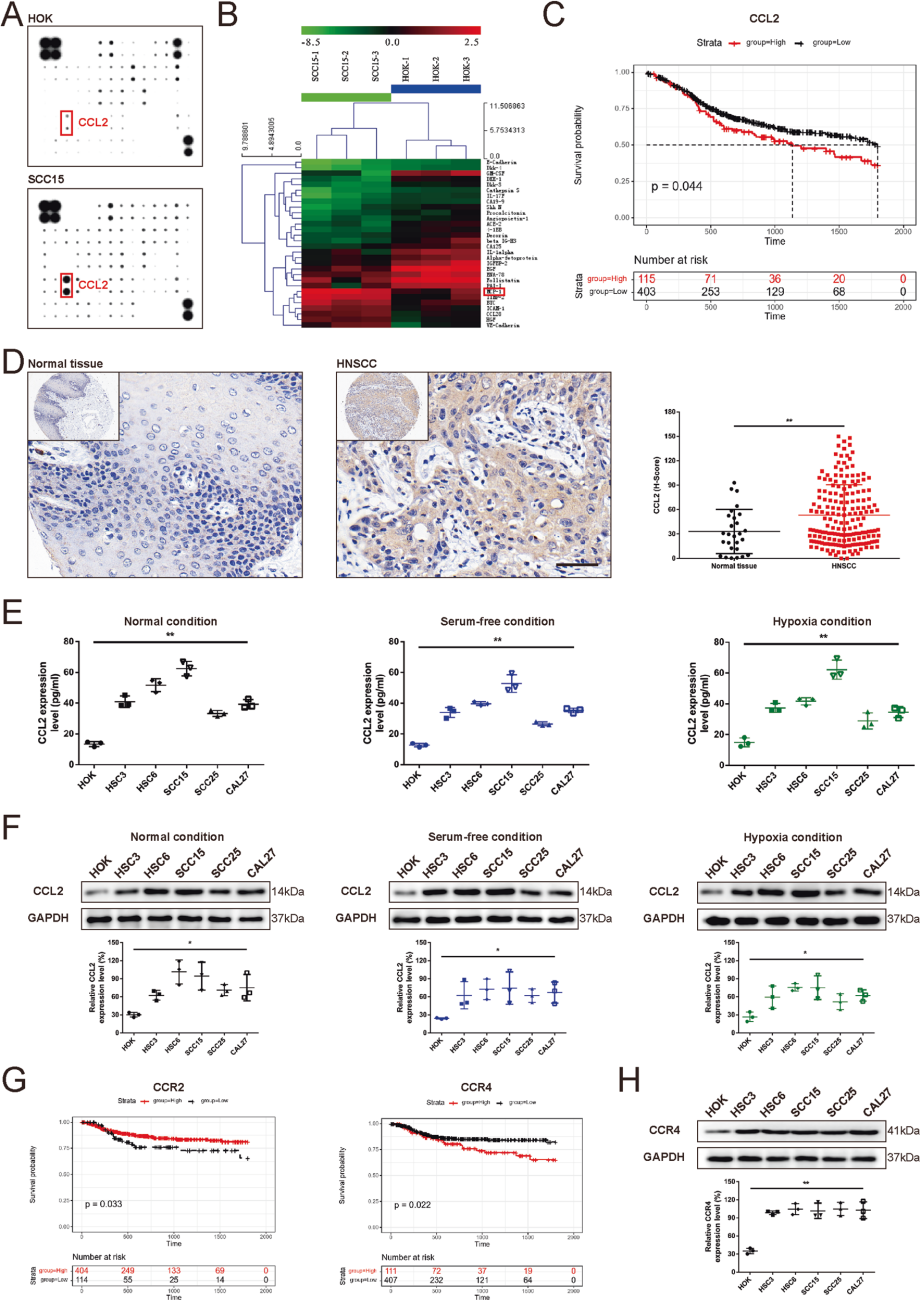
Overexpression of CCL2 was inherent and predicted poor prognosis in HNSCC. **A** The representative images of chip detection. RayBio human inflammatory cytokine antibody array was used to screen the expression profiles of chemokine and cytokine in cells culture supernatants of HNSCC cells grown in serum-free medium for 24 h. **B** The representative heatmaps of differentially expressed cytokines in HNSCC cells compared with the HOK (Green: downregulated, Red: upregulated). CCL2 (MCP-1) is one of the overexpressed chemokines in the supernatants of HNSCC cells compared with HOK cells. **C** Kaplan–Meier survival curves of HNSCC patients with low and high CCL2 expression. Kaplan–Meier curves for disease-specific survival (DSS) in 518 HNSCC patients, which were classified by the relative (high or low) immune signal for CCL2 protein levels, respectively. The log-rank (Mantel–Cox) test *p* value reflects the significance of the correlation between lower CCL2 expression and longer survival outcomes. **D** Overexpression of CCL2 in HNSCC tissue. The IHC staining revealed that CCL2 expression in HNSCC tissues (*n* = 180) was significantly higher than that of cancer adjacent tissue (*n* = 27). (Magnification, ×400; Bar: 50 μm; **P < 0.01). **E** High CCL2 expression of HNSCC cells was inherent but not attributed to the deprivation of serum or oxygen. ELISA assays revealed that the CCL2 level of cultured supernatants in HNSCC cells was significantly higher than that of HOK cells and not significantly related to the amount of fetal bovine serum or oxygen supply (**P < 0.01). **F** Inherent high CCL2 expression of HNSCC cells were confirmed by Western blot (*P < 0.05). **G** Kaplan–Meier survival curves of HNSCC patients with low and high CCR2 or CCR4 expression. Kaplan–Meier curves for disease-specific survival (DSS) in 518 HNSCC patients, which were classified by the relative (high or low) immune signal for CCR2 or CCR4 protein levels, respectively. The log-rank (Mantel–Cox) test *p* value reflects the significance that lower CCR2 or higher CCR4 expression was correlated with longer survival outcomes. **H** High CCR4 expression of HNSCC cells were confirmed by Western blot (**P < 0.01).

Total 32 differential proteins were identified in SCC15 cells compared with human oral keratinocyte (HOK) cells in serum-deficient conditions (Supplementary Table 1). CCL2 was a highly expressed cytokine in HNSCC cells under the serum-deficient condition (Fig. 1A, B), indicating that tumor cells were an important cellular source of CCL2 in the tumor microenvironment. Kaplan–Meier analysis of specimens from HNSCC patients revealed that patients with low CCL2 protein levels had longer disease-specific survival (DSS) than patients with high CCL2 protein levels (Fig. 1C). It indicated that high expression of CCL2 in HNSCC predicts a relatively shorter survival period, which may associate with promoted tumor progression activated by excessive CCL2.

In order to verify the above speculation, we further determined the expression level of CCL2 in HNSCC tissues and more HNSCC cell lines. Sure enough, the result of IHC staining revealed that CCL2 expression in HNSCC tissues was significantly higher than that of cancer adjacent tissue (Fig. 1D and Supplementary Table 2). Next, we confirmed the overexpression of CCL2 in HNSCC cells by ELISA (Fig. 1E) and Western blot (Fig. 1F). Moreover, CCL2 overexpression in HNSCC cells was inherent because high CCL2 expression remains unaffected by hypoxia conditions (1% oxygen concentration) and serum-free condition in vitro (Fig. 1E,F).

As a chemokine, CCL2 should bind to its receptors to play related molecular functions. Therefore, we focused on the two significant receptors of CCL2, CCR2, and CCR4, and revealed the correlation between the expression levels of these two receptors and the DSS of HNSCC patients through Kaplan–Meier analysis, respectively. Unexpectedly, the expression level of CCR2, the widely concerned classical receptor for CCL2, was positively correlated with DSS in HNSCC. While the higher expression level of CCR4 is correlated with the poor prognosis of HNSCC patients (Fig. 1G). This indicated that the CCL2-CCR4 axis may play a more important role in the progress of HNSCC than the CCL2-CCR2 axis. We determined the protein level of CCR4 in HOK and HNSCC cells under normal culture conditions, and the results showed that the expression level of CCR4 in HNSCC cells was higher than that in HOK cells, but the expression level of CCR4 in several HNSCC cell lines selected in this study is relatively close (Fig. 1H). Therefore, we selected the HSC6 cell line and SCC15 cell line in subsequent in vitro experiments due to their higher expression level of CCL2.

### CCL2 promote the motility of HNSCC cells through CCR4 in vitro and in vivo

**Fig. 2:**
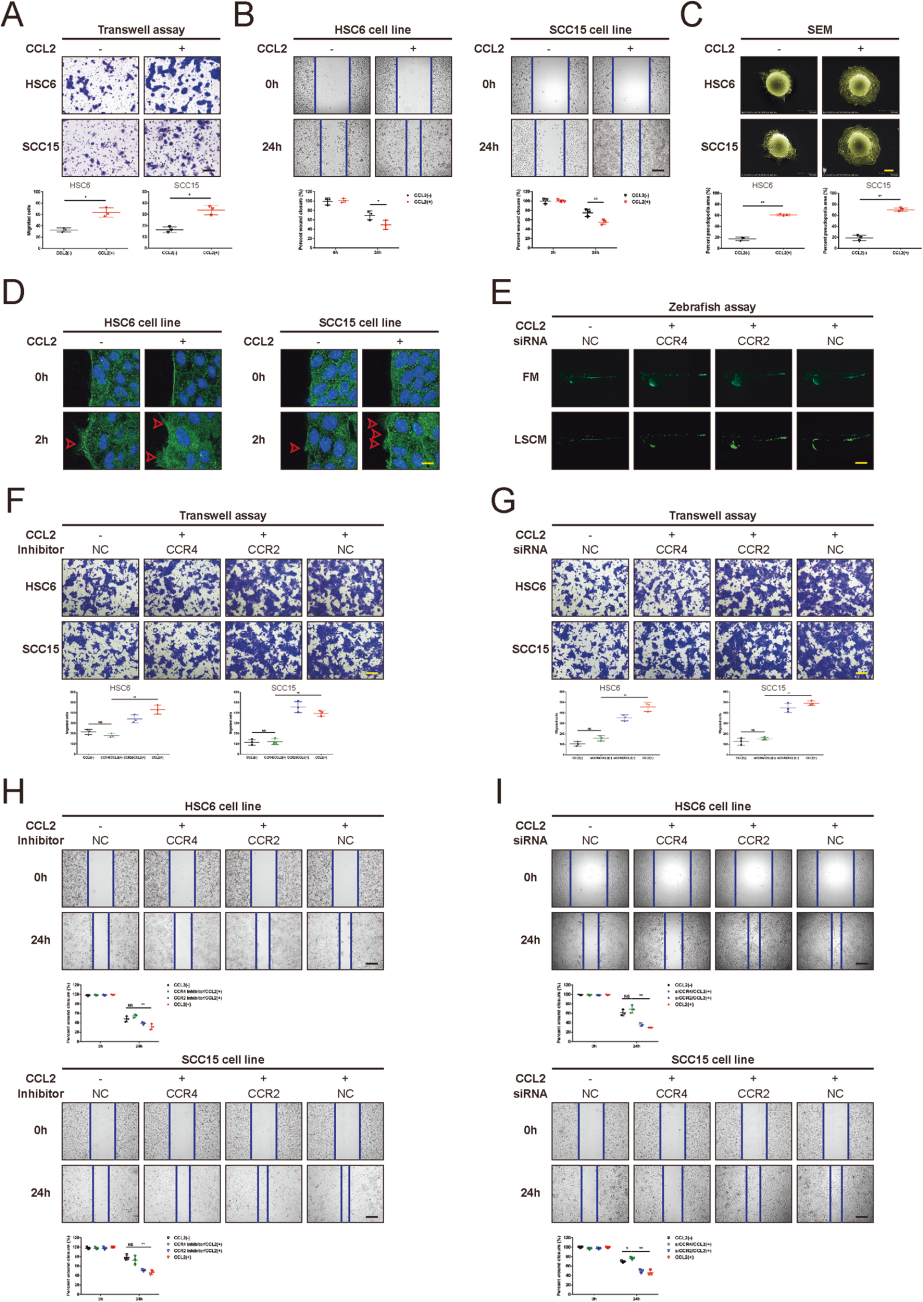
CCL2 promotes the motility of HNSCC cells through CCR4 in vitro and in vivo. **A**, **B** Transwell migration assays (**A**) and wound healing assays (**B**) were performed in HNSCC cells cultured with or without exogenous CCL2 (100 ng/mL) for 24 h. As shown in representative images, CCL2 could significantly induce the migration of HNSCC cells. (Fig. 2A, Magnification, ×100; Bar: 200 μm; Fig. 2B, Magnification, ×50; Bar: 400 μm; *P < 0.05; **P < 0.01). **C** SEM was used to detect the pseudopodia in HNSCC cells treated with CCL2 (100 ng/mL) for 2 h. Data of percent pseudopodia area were analyzed using ImageJ (Magnification, ×3000; Bar: 5 μm; **P < 0.01). **D** Confocal images showed the increased cytoskeleton remodeling in HNSCC cells induced by CCL2 (100 ng/mL) for 2 h (Original magnification, ×630; Bar: 20 μm; red arrows, stained with green fluorescence for β-actin and blue fluorescence for DAPI). **E** CCL2 promoted cell motility and distant metastasis of HNSCC cells in zebrafish, but the effect was abolished with siCCR4. HNSCC cells were labeled by GFP and implanted into the zebrafish embryos. After implantation, the embryos were monitored using fluorescence microscopy and LSCM for 3 days to monitor the migration of implanted cells. (Original magnification, ×50; Bar: 1000 μm). **F**–**I** Transwell migration assays (**F**, **G**) and wound healing assays (**H**, **I**) were performed in HNSCC cells cultured with CCR4 inhibitor (100 nM) and CCR2 inhibitor (20 μM), or siCCR4 and siCCR2, respectively (Fig. 2F, G, Magnification, ×100; Bar: 200 μm; Fig. 2H, I, Magnification, ×50; Bar: 400 μm; *P < 0.05; **P < 0.01; NS no statistical significance).

According to our preliminary research, there was no significant effect on cell proliferation of HNSCC cells treated with CCL2 in vitro. However, we found that HNSCC cells treated with exogenous CCL2 (100 ng/ml) exhibited much better motility compared to controls (Fig. 2A). To confirm the Transwell migration data, we performed the wound healing assay to assess cell capacity to migrate and repair the wound. As illustrated in Fig. 2B, both HSC6 and SCC15 cells become more motile after being treated with CCL2. Notably, cytoskeleton remodeling and filopodia are crucial for cell motility. We observed that the surface of tumor cells exhibited more numerous and longer filopodia after treatment with CCL2 for 2 hs (Fig. 2C). Next, we performed the immunofluorescence staining of β-Actin to access the morphologic changes in HNSCC cells treated with CCL2. The results indicated more spike-like protrusions in CCL2-treated cells extended initially compared with controls (Fig. 2D). These new-formed protrusions at the leading edge facilitated the efficient migration of HNSCC cells (Fig. 2D).

In order to explore the function of CCL2 in the development of HNSCC in vivo, the zebrafish model was used. As depicted in Fig. 2E, GFP-labeled SCC15 cells survived and remained visible for 3 days post-injection in vivo experiment. We observed that CCL2 seemingly didn’t significantly promote the proliferation of HNSCC cells but promoted the dissemination of injected cells to extravasate and engraft mainly in the perivascular milieu of caudal hematopoietic tissues, revealing a migratory phenotype. More importantly, the interference of CCR4 by siRNA could significantly inhibit the migration ability of cells induced by CCL2 in the zebrafish model.

To decipher the molecular mechanism as to how CCL2 enhances HNSCC cell migration, we have intervened in CCR2 and CCR4, which were reported to be associated with cancer cell progression. We used CCR2 specific antagonists (20 μM, RS102895 hydrochloride, MedChemExpress, Monmouth Junction, NJ) and CCR4 specific antagonists (100 nM, AZD2098, MedChemExpress, Monmouth Junction, NJ) or silencing short interfering RNA to block CCR2 and CCR4 in HSC6 cells and SCC15 cells, and results revealed that suppression of CCR4 instead of CCR2, could reverse CCL2-promoted HNSCC cells migration in both Transwell assay and wound healing (Fig. 2F–I). The sequences and knockdown efficiency of each siRNA in HNSCC cells were represented in Supplementary Table 3 and Supplementary Fig. 1. Moreover, ELISA assay and qRT-PCR demonstrated that CCL2 did not upregulate the levels of other functional ligands of CCR4, including CCL17 or CCL22, in HNSCC (Supplementary Fig. 2). Primers used for qRT-PCR were listed in Supplementary Table 4.

### CCL2 enhances HNSCC cell motility via promoting Rac1- phosphorylated MLC (p-MLC) activation

**Fig. 3:**
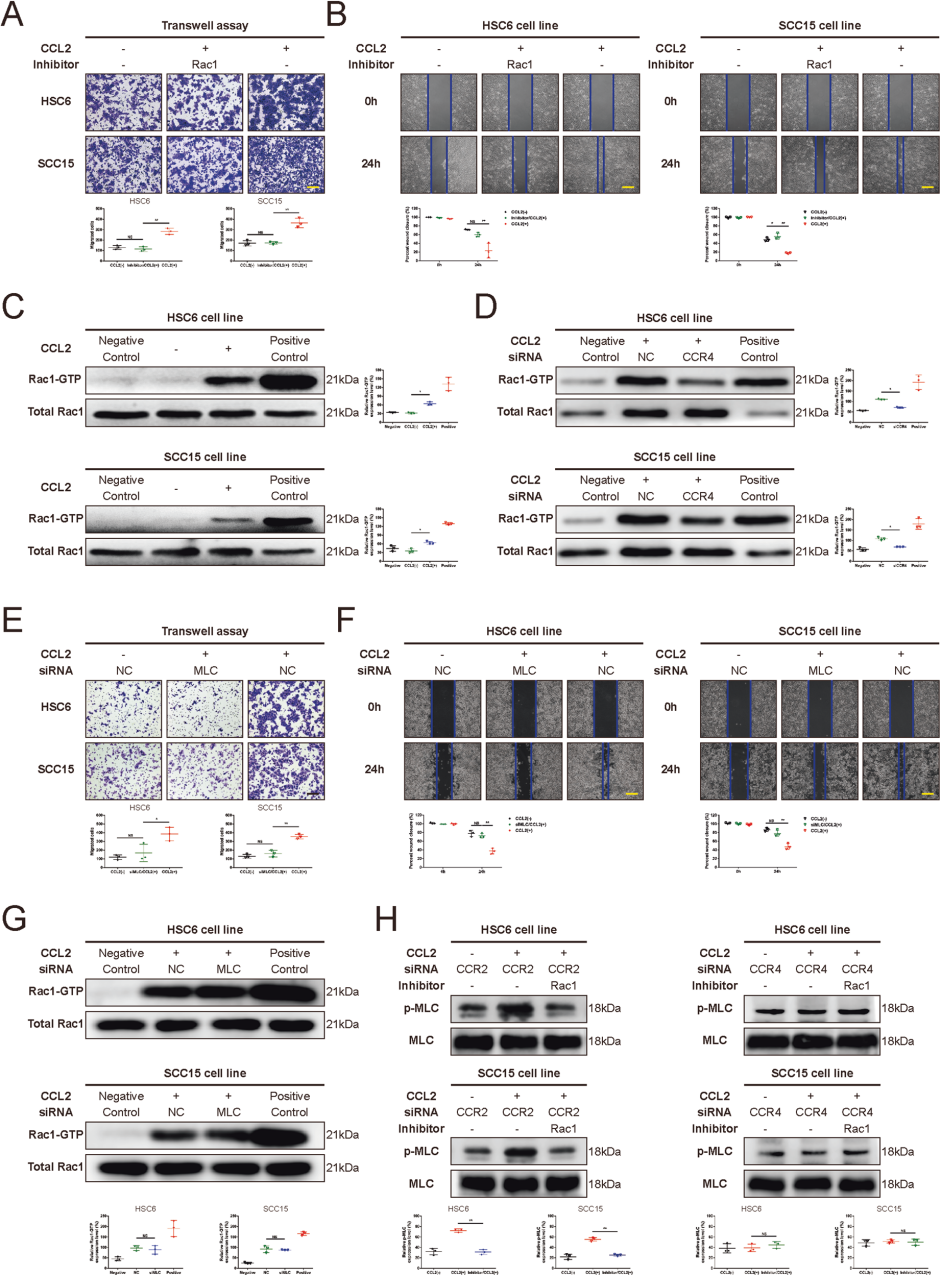
The crucial role of Rac1-p-MLC activation in the CCL2-CCR4 axis induced HNSCC cell migration. **A**, **B** Rac1 inhibitor could restrain the migration induced by CCL2 in HNSCC cells. Transwell migration assay (**A**) and wound healing assay (**B**) were performed in HNSCC cells cultured with or without Rac1 inhibitor (100 μM). The migration of HNSCC cells treated with Rac1 inhibitor significantly decreased compared with controls (Fig. 3A, Magnification, ×100; Bar: 200 μm; Fig. 3B, Magnification, ×50; Bar: 400 μm; *P < 0.05; **P < 0.01; NS no statistical significance). **C** CCL2 upregulated the activation of Rac1. The Thermo Scientific Active Rac1 Pull-Down and Detection Kit were used to detect the GTP-bound Rac1, and results indicated that the amount of GTP-bound Rac1 was significantly increased in HNSCC cells treated with CCL2 (*P < 0.05). **D** siCCR4 could inhibit the activation of Rac1 induced by CCL2. The amount of GTP-bound Rac1 in HNSCC cells transfected with siCCR4 was detected and compared with that of the NC group. Results revealed that CCR4 inhibition could abolish the upregulation of GTP-bound Rac1 induced by CCL2 (*P < 0.05). **E**, **F** MLC were essential for CCL2-induced HNSCC cell migration. Transwell migration assay (**E**) and wound healing assay (**F**) were performed in HNSCC cells with siMLC compared with the NC group. The migration of HNSCC cells transfected with siMLC significantly reduced compared with NC group (Fig. 3E, Magnification, ×100; Bar: 200 μm; Fig. 3F, Magnification, ×50; Bar: 400 μm; *P < 0.05; **P < 0.01; NS no statistical significance). **G** MLC was a downstream target of Rac1. The amount of GTP-bound Rac1 in HNSCC cells transfected with siMLC was detected and compared with that of the NC group. There was no statistical significance in the amount of GTP-bound Rac1 between HNSCC cells with the siMLC group and the NC group. It suggested that MLC was a downstream target of Rac1 (NS no statistical significance). **H** CCL2-CCR4 signaling induced the upregulation of p-MLC through activation of Rac1. The levels of p-MLC of HNSCC cells with siCCR2 or siCCR4 were detected and compared after culturing with or without Rac1 inhibitor (100 μM). Results indicated that CCR4 (not CCR2) inhibition could significantly suppress the upregulation of p-MLC in HNSCC cells induced by CCL2. Moreover, results confirmed the crucial role of Rac1 in the upregulation of p-MLC induced by CCL2-CCR4 signaling. (**P < 0.01; NS no statistical significance).

Given that the Rac1-p-MLC signal had been reported to play an important role in cell motility by promoting the contractile motion of the myosin light chain, we used interruption approaches with a specific inhibitor to block Rac1 (100 μM, NSC 23766, MedChemExpress, Monmouth Junction, NJ), we found that the cell migration was suppressed in HNSCC cells treated with CCL2 (Fig. 3A, B). Then we performed the pulldown assay to access Rac1 and found the overexpression of GTP-bound Rac1 in HNSCC cells after treatment with CCL2 but not overexpression of total Rac1 (Fig. 3C). Interestingly, we also found that the increase of GTP-bound Rac1 induced by CCL2 could be blocked with siCCR4, but not with siCCR2, in HNSCC cells (Fig. 3D and Supplementary Fig. 3). Next, we used a specific siRNA to knock down MLC and found that cell migration was not improved in HNSCC cells treated with CCL2 (Fig. 3E, F) although the level of GTP-bound Rac1 was upregulated (Fig. 3G). Moreover, the phosphorylation of MLC was reversed by the Rac1 inhibitor in a CCR4-dependant manner in HNSCC cells treated with CCL2 (Fig. 3H and Supplementary Fig. 4). Taken together, these results recognized the Rac1-p-MLC signal as the downstream of the pathway of CCL2-CCR4 could promote the HNSCC cell migration. The sequences and knockdown efficiency of each siRNA in HNSCC cells were represented in Supplementary Table 3 and Supplementary Fig. 1.

### Vav2 is required for Rac1 activation induced by CCL2-CCR4 signaling

**Fig. 4:**
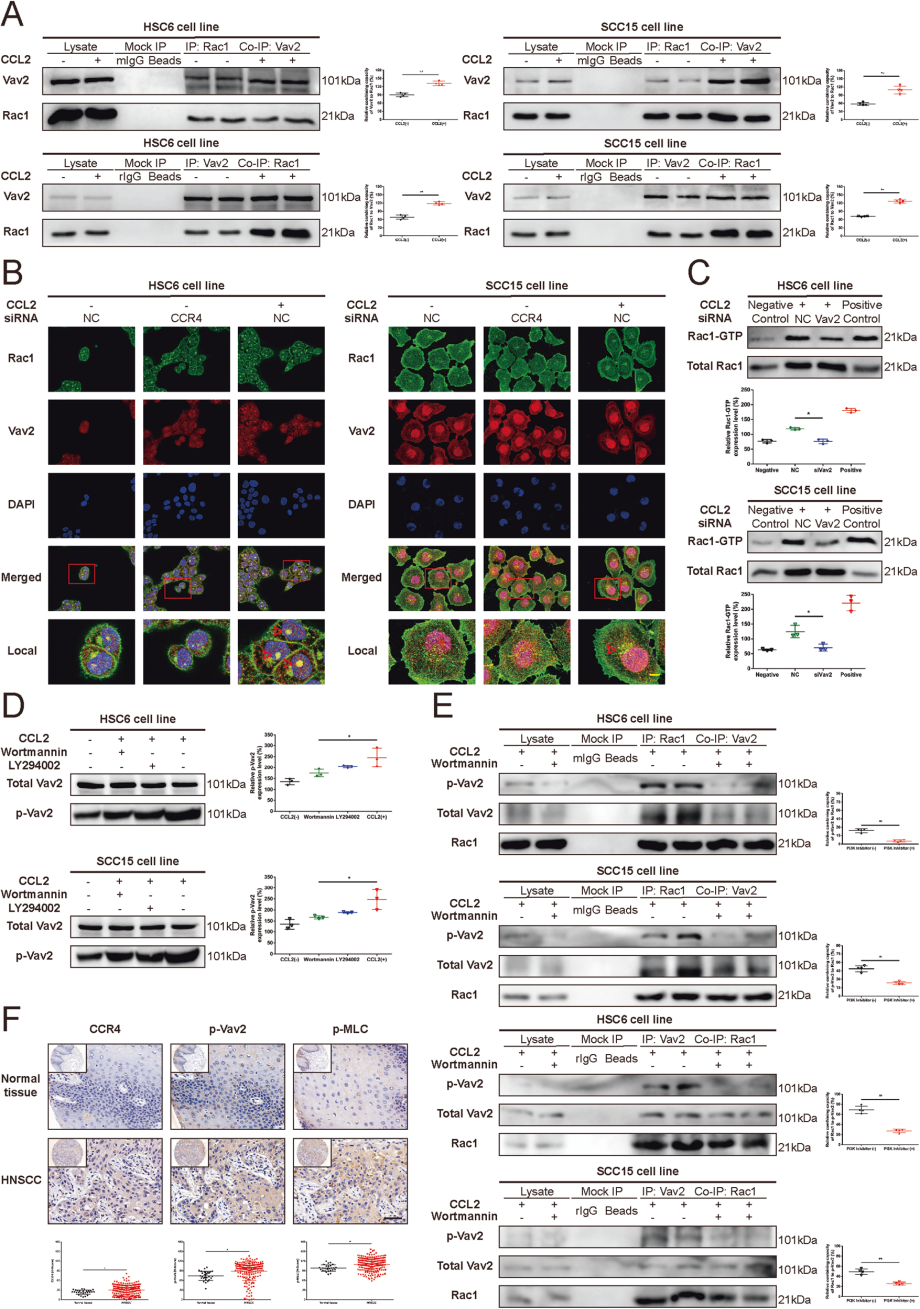
Vav2 is essential for CCL2-CCR4 signaling induced Rac1 activation and cell migration. **A** CCL2 stimulated the formation of the Vav2-Rac1 complex. HNSCC cells were subjected to treatment with CCL2 for 2 h. The cell lysates were subjected to immunoprecipitation (IP) with Rac1 or Vav2 antibodies, respectively. The immunoprecipitates were subjected to Western blot assay with the indicated antibodies. Results revealed that CCL2 did not upregulate the expression of Vav2 or Rac1 but significantly induce the Vav2-Rac-1 complex formation in HNSCC cells treated with CCL2 (**P < 0.01). **B** CCL2 stimulated the colocalization of Vav2 and Rac1 via CCR4. HNSCC cells were treated with or without exogenous CCL2 (100 ng/mL) for 2 h and stained with green fluorescence (for Rac1), red fluorescence (for Vav2), and DAPI (nuclear stain). Confocal images showed that CCL2 promoted the colocalization of Vav2 and Rac1 in the cytoplasm (red arrows); however, it was abolished with siCCR4 (Original magnification, ×400; Bar: 5 μm). **C** Vav2-silencing short interfering RNA (siVav2) could inhibit the activation of Rac1 induced by CCL2. HNSCC cells were either untreated or treated with negative control siRNA or siVav2 for 48 h. The cells were then treated with exogenous CCL2 (100 ng/mL) for 2 h. The amount of GTP-bound Rac1 was significantly reduced in HNSCC cells transfected with siVav2 compared with the NC group (*P < 0.05). **D** CCL2 induced the upregulation of p-Vav2 through PI3K signaling. HNSCC cells were incubated in a culture medium with or without PI3K inhibitors (LY294002, 50 μM; Wortmannin 2 μM, respectively) for 24 h. The cells were then treated with exogenous CCL2 (100 ng/mL) for 2 h. The cell lysates were subjected to Western blot assay with antibodies against p-Vav2 and total Vav2. Results revealed that PI3K inhibition could inhibit the upregulation of p-Vav2 induced by CCL2 (*P < 0.05). **E** CCL2 induced the formation of the Vav2-Rac1 complex in a PI3K-dependent manner. HNSCC cells were incubated in a culture medium with or without the PI3K inhibitor (Wortmannin, 2 μM) for 24 h. The cells were then treated with exogenous CCL2 (100 ng/mL) for 2 h. HNSCC cell lysates were subjected to immunoprecipitation (IP) with Rac1 or Vav2 antibodies, respectively. The immunoprecipitates were subjected to Western blot assay with the indicated antibodies. Results revealed that CCL2 upregulates the phosphorylation level of Vav2 and significantly induces the Vav2-Rac1 complex formation in HNSCC cells. But the PI3K inhibitor could abolish the enhancement of Vav2-Rac1 complex formation and phosphorylation level of Vav2 induced by CCL2 (**P < 0.01). **F** Differential expression of CCR4, p-Vav2, and p-MLC between normal tissue and HNSCC tissue. IHC staining was used to detect the presence of CCR4, p-Vav2, and p-MLC expression in normal tissue and HNSCC tissue. The results revealed that expression of CCR4, p-Vav2, and p-MLC was significant higher in HNSCC tissues compared with normal tissues (Magnification, ×400; Bar: 50 μm; *P < 0.05; **P < 0.01).

We first detected the mRNA expression of Prex1, Prex2, Vav2, Vav3, and ECT2 in HNSCC cells by qRT-PCR (Supplementary Fig. 5). To confirm which GEF might be involved in Rac1 activation downstream from stimulation of HNSCC cells, we performed a Co-IP assay in the present study. We found that CCL2 treatment for 2 h increased the association of Vav2 with Rac1 (Fig. 4A). However, Rac1 failed to increase the affinity of Prex1 or ECT2 bond small GTPases after CCL2 treatment in HNSCC cells (Supplementary Fig. 6). We then observe the colocalization of Vav2 and Rac1 in the cytoplasm after CCL2 treatment in HNSCC cells. The result indicated that CCL2 could stimulate the colocalization of Vav2 and Rac1 in the cytoplasm in a CCR4-dependent manner, demonstrating the findings that silencing of CCR4 with RNAi perturbs CCL2-stimulated Vav2 binding with Rac1 to form the Vav2-Rac1 complex (Fig. 4B).

To further study the role of Vav2 in CCL2-induced Rac1 activation, we used siRNA against Vav2 to downregulate Vav2 expression in HNSCC cells. Following the knockdown of Vav2 expression, there was an impaired Rac1 activation in response to CCL2 in HNSCC cells, demonstrating the downregulation of GTP-bound Rac1 (Fig. 4C). Moreover, we found that activation of Vav2 tyrosine phosphorylation induced by CCL2 was partly reversed by two PI3K-AKT pathway inhibitors, Wortmannin (2 μM, MedChemExpress, Monmouth Junction, NJ) and LY294002 (50 μM, MedChemExpress, Monmouth Junction, NJ) but did not affect by the Src inhibitor PP2 (1 μM, MedChemExpress, Monmouth Junction, NJ) in HNSCC cells (Fig. 4D, E and Supplementary Fig. 7). Therefore, CCL2 promoted the formation of Vav2-Rac1 by inducing the Vav2 phosphorylation through the activation of the PI3k-AKT pathway. H-Score analysis also revealed that the expression levels of CCR4, p-Vav2, and p-MLC in HNSCC tissues were significantly higher than those in normal tissues (Fig. 4F) which further verified the above results. The details of the human tissue microarrays OR208 containing patient samples were shown in Supplementary Table 2.

### CCR4 antagonist inhibited CCL2-mediated HNSCC cells migration and invasion in the in vivo xenograft model

**Fig. 5:**
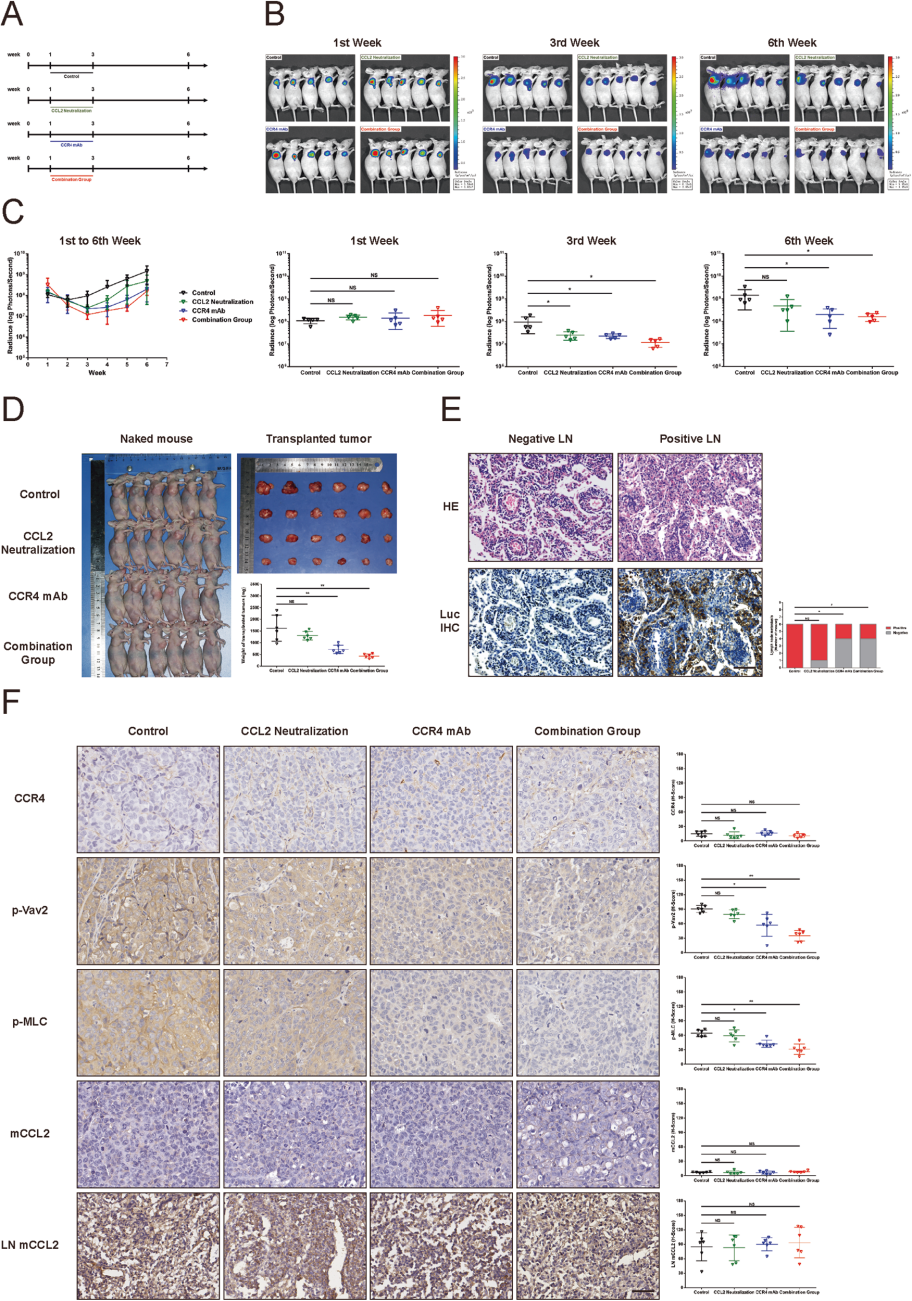
CCR4 monoclonal antibody blocks the activation of Vav2-Rac1-MLC signaling induced by CCL2 and inhibits HNSCC growth and invasion in vivo. **A** Experimental design of nude mice in vivo. Twenty-four nude mice were randomly divided into four groups, with six mice in each group. Each nude mouse was injected with 2.5 million SCC15 cells labeled with luciferase under the skin of the axillary for subcutaneous tumor formation (0 W). In 1–3 weeks, four groups of nude mice were treated as indicated in the flow chart, respectively. All treatments were terminated at 3–6 weeks, and all mice were euthanized at the 7th week. The axillary lymph nodes and tumors were collected for IHC staining and weight measurement. **B**, **C** Biofluorescence analysis of nude mice. Bioluminescence images were obtained once a week to analyze the tumor growth and local invasion from 1st to the 6th week. There was no significant difference in tumor size among the four groups before treatment (1st week). After 2 weeks of treatment, tumor sizes of three treatment groups were significantly smaller than that of the control group (3rd week). With the suspension of treatment for 3 weeks, the tumor sizes of the CCL2 neutralizing antibody group progressed particularly rapidly, even approaching to control group. However, the tumor progression in the CCR4 monoclonal antibody group and combination group were flatted and significantly slower than that of tumors of the control group (6th weeks) (*P < 0.05; NS no statistical significance). **D** CCR4 monoclonal antibody inhibited HNSCC progression without rapid relapse after treatment withdrawal. All mice were euthanized at the 7th week, and the tumor sizes were measured. It was similar to the results of the bioluminescence analysis; the tumor weight in the CCL2 neutralizing antibody group exhibited no significant difference between that of the control group. But the tumor weight of both the CCR4 monoclonal antibody group and combination group was lighter than that of the control group (**P < 0.01; NS no statistical significance). **E** CCR4 monoclonal antibody inhibited the lymph node metastasis of HNSCC. Representative images of HE and Luciferase staining (Luc IHC) in the axillary lymph nodes. The number of metastatic lymph nodes (Positive LN) of both the CCR4 monoclonal antibody group and combination group was lesser than that of the control group, but the CCL2 neutralizing antibody group was an exception (Original magnification, ×400; Bar: 20 μm; *P < 0.05; NS no statistical significance). **F** CCR4 monoclonal antibody inhibited the activation of Vav2-Rac1-MLC signaling in HNSCC. Representative images of CCR4, p-Vav2, and p-MLC staining in the xenograft tumors. Although there was no significant difference in CCR4 staining in each group, p-Vav2 and p-MLC level of both the CCR4 monoclonal antibody group and combination group were weaker than that of the CCL2 neutralizing antibody group and control group. In addition, the expression levels of mouse CCL2 (mCCL2) in the transplanted tumors and mouse lymph nodes (LN) of each group were not statistically different, it was considered that the expression of mCCL2 would not affect the mobility of HNSCC cells in the in vivo xenograft model. (Magnification, ×400; Bar: 50 μm; *P < 0.05; NS no statistical significance).

To confirm the CCL2-induced HNSCC cell migration and invasion in vivo, we transduced SCC15 cells with lentiviral-luciferase plasmid and selectively expanded the positive stable cells. The mice were randomly divided into four groups as indicated in Fig. 5A. Tumor progression was monitored in live animals using in vivo imaging system every week. The results revealed that SCC15 cells implanted in mice were treated with CCL2 neutralizing antibody, CCR4 antagonist Mogamulizumab, and the combination injection exhibited lower luminescence signals located at local sites compared to the normal saline (NS) (Fig. 5B, C). Unsurprisingly, we also found that the treatment with CCR4 antagonist or the combination injection had similar inhibition in growth rates compared to treatment with CCL2 neutralizing antibody during treatment (Fig. 5B, C).

In order to determine the relapse and metastasis of the tumor, the treatment was terminated for 3 weeks. The results revealed that cessation of CCL2 neutralizing antibody caused a more repaid recurrence of HNSCC compared to the cessation of CCR4 antagonist (Fig. 5B–D). Moreover, there was no statistical difference in tumor sizes between the CCR4 antagonist and the combination groups after the cessation of treatment for 3 weeks (Fig. 5B–D). However, CCL2 and CCR4 antagonist did not significantly change the cell cycle and proliferation of HNSCC cells (Supplementary Fig. 8).

For tumor cell metastasis, the CCR4 antagonist reduced the lymph node metastasis more effectively than the CCL2 neutralizing antibody (Fig. 5E). As anticipated, H&E and IHC staining of those metastatic foci confirmed micrometastases in HNSCC cells (Fig. 5E). At the same time, IHC staining in transplanted tumors indicated that the CCR4 antagonist could effectively downregulate the phosphorylation of Vav2 and MLC in cancer cells, but not in tumors treated with CCL2 neutralizing antibody (Fig. 5F). Since the expression levels of mouse CCL2 (mCCL2) in the transplanted tumors and mouse lymph nodes of each group were not statistically different, it was considered that the expression of mCCL2 would not affect the mobility of HNSCC cells in the in vivo xenograft model (Fig. 5F).

**Fig. 6:**
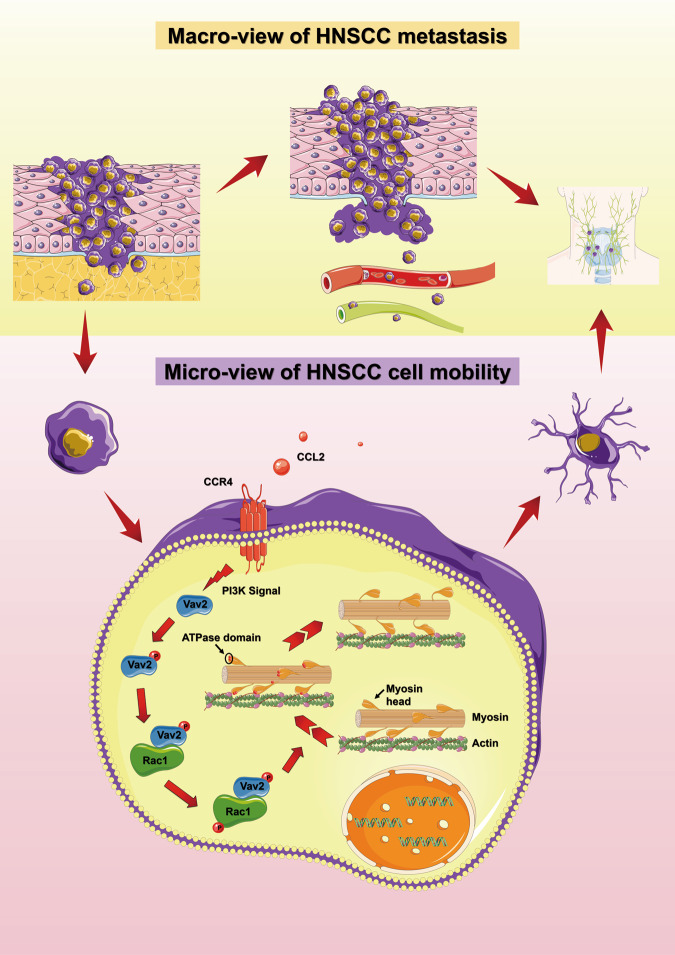
The proposed mechanism of how CCL2 enhances cell motility of HNSCC. The proposed models described the effect of CCL2 on cell migration and tumor metastasis in HNSCC. From a macroscopic perspective, HNSCC can metastasize to distant sites through lymphatics and blood vessels, among which cervical lymph node metastasis was most frequent. From a microscopic perspective, HNSCC cells can secrete CCL2 which could bind to the membrane receptor CCR4 by autocrine or paracrine function. Then, the PI3K pathway transduces extracellular signals into the cell and activates the Vav2-Rac1-MLC signal axis. Finally, CCL2 promotes HNSCC cytoskeletal remodeling and pseudopodia formation, enhances the mobility of HNSCC cells, and promotes the metastasis of HNSCC.

Together, all these results suggested that CCL2 promoted the formation of the Vav2-Rac1 complex to activate Rac1 and then induce cell migration via CCR4. A proposed model for the regulatory role CCL2 of HNSCC cell motility was schematically summarized in Fig. 6.

## Discussion

Although an increasing number of studies have shown that CCL2-CCR2/CCR4 axis plays crucial role in the progression and metastasis of multiple solid tumors [5], the exact molecular mechanisms of this axis on HNSCC and its clinical implications remain elusive. In the present study, we demonstrated that the CCL2-CCR4 axis, not CCL2-CCR2 signaling, mediates the CCL2-induced migration and invasion of human HNSCC cells and targeted inhibition of CCR4 significantly inhibit the invasion and metastasis of HNSCC xenografts in the nude mouse without causing relapse during the cessation of CCL2 antibody therapy.

Previous studies have reported that overexpression of CCL2 and its receptors, CCR2, and CCR4, in human cancers, promote lung cancer, breast cancer, and HNSCC development via regulation of angiogenesis, cell proliferation, and migration [5, 7–9, 24, 25]. All these findings suggested that CCL2 is involved in carcinogenesis and tumor progression. However, which receptor, CCR2 or CCR4, plays a critical role in activating downstream signaling molecules of CCL2 remains obscure. Our study identified that CCL2 promoted cell migration of HNSCC cells via CCR4, not by CCR2. Furthermore, we identified that CCL2 and CCR4 was highly expressed in cancer cells of HNSCC, indicating that CCL2 secreted by cancer cells possibly stimulates itself by autocrine or paracrine function. Together, these results suggested that CCL2-CCR4 signaling played an essential role in the local invasion and metastasis of HNSCC, and CCR4 might serve as a potential therapeutic molecular target by inhibiting tumor invasion metastasis [26].

Rac1, a member of the Rac subfamily of Rho-GTPases, is a pleiotropic regulator of multiple cellular processes, including cell motility [27–29]. Vav2 had been reported to upregulate cell motility by promoting the cycling between an inactive Rac1-GDP-bound state to an active Rac1-GTP-bound state in many cancer types [30–32]. However, whether Vav2-Rac1 may exert their pleiotropic effects on cancer cells treated with CCL2 as a complex remains unclear. Our findings that the pharmacological inhibition and genetic suppression of Vav2 also inhibited the CCL2-mediated activation of Rac1 in human HNSCC cells in a CCR4-dependent manner, indicating that Vav2 plays a critical role in the activation of Rac1 in HNSCC cells treated with CCL2. Moreover, CO-IP and confocal microscopy results revealed that the binding of Vav2 and Rac1 was enhanced in cancer cells following treatment with CCL2, which indicated that CCL2 induces the functional coupling of Vav2 and Rac1 and the formation of the Vav2-Rac1 molecular complex.

Next, we investigated the downstream target genes of Rac1 regulating cell motility. As previously reported, the remodeling of cytoskeleton and myosin contraction is crucial for cell movement [33]. Studies have shown that several factors, including Rac1, can promote the phosphorylation of MLC protein [28, 34, 35]. However, whether CCR4 can promote MLC phosphorylation through Vav2/Rac1 signaling pathway and induce HNSCC migration has not been reported. In this study, we observed that the CCL2-CCR4 axis could effectively induce the activation of Rac1 and increase the activation of MLC phosphorylation in HNSCC cells. Moreover, our results indicated that Rac1 inhibitors could effectively inhibit CCL2-CCR4 induced Rac1 activation and the MLC phosphorylation. More importantly, we observed that MLC inhibitor could not block the activation of Rac1 induced by CCL2-CCR4; however, it could inhibit the phosphorylation of MLC and suppress cell migration in HNSCC cells. These findings collectively indicated that MLC is the main downstream target of Vav2-Rac1 signaling and plays a critical role in cell migration in HNSCC cells treated with CCL2. The present study reported that CCR4-Vav2-Rac1-MLC signaling participates in cell migration in HNSCC.

To verify the therapeutic potential of CCL2-CCR4 signaling in a solid tumor, in this study, we used Mogamulizumab, a humanized anti-CCR4 monoclonal antibody, a promising agent for CCR4-positive T-cell lymphomas [36], to treat subcutaneously implanted HNSCC tumor in nude mice. Our results showed that Mogamulizumab could effectively inhibit the local invasion and the metastasis of distant lymph nodes by blocking CCL2-CCR4-Vav2-Rac1-MLC signaling in the HNSCC xenograft model. Because a previous study reported that the suspension of CCL2 neutralizing antibody therapy could lead to rapid tumor recurrence due to monocyte release from the bone marrow and blood vessel formation [15], we also discontinued Mogamulizumab therapy to investigate the recurrence of tumor in this study. As anticipated, we observed the slow recurrence of implanted tumors after cessation of Mogamulizumab and CCL2 neutralizing antibody therapy, respectively. However, we did not observe abundant monocytes’ infiltration and highly active angiogenesis in tumor lesions after discontinuation of Mogamulizumab therapy. It appeared reasonable that the recurrence of tumors after suspension of Mogamulizumab therapy was slower than those with discontinued CCL2 neutralizing antibody therapy. Therefore, CCR4 represents an effective target for inhibiting local invasion and distant metastasis in HNSCC and circumventing the rebound phenomenon that might occur during the cessation or interruption of CCL2 neutralizing antibody therapy.

In summary, the present study has identified and confirmed that CCL2 could enhance HNSCC cell metastasis via activation of the CCR4-Vav2-Rac1-MLC signaling axis. Besides, targeting CCR4 to block this newly identified signaling may help in the development of alternative new strategies for suppression of HNSCC tumor metastasis.

## Materials and methods

### Cell culture

The human head and neck squamous cell carcinoma (HNSCC) cell line HSC3, HSC6 were provided by J. Silvio Gutkind (NIH, Bethesda, MD, USA). Human HNSCC cell lines SCC15, SCC25, and CAL27, human embryonic kidney cell lines HEK-293, human oral keratinocyte (HOK) were procured from the American Type Culture Collection (ATCC; Rockville, MD, USA). HSC3, HSC6, CAL27, and HEK-293 were cultured in Dulbecco’s modified Eagle medium (DMEM; Gibco, Rockville, MD, USA) supplemented with 10% fetal bovine serum (FBS; Invitrogen, Carlsbad, CA, USA). SCC15 and SCC25 cells were maintained in DMEM/F12 (1:1) (Gibco, Rockville, MD, USA) supplemented with 10% FBS. HOK cells were cultured in keratinocyte serum-free medium (KSFM; Invitrogen) supplemented with 5 ng/ml epidermal growth factor (EGF) and 50 μg/mL of bovine pituitary extract (GIBCO). All cells were incubated in a humidified atmosphere containing 5% CO_2_ at 37 °C.

### Human cytokine antibody array

Culture supernatant was used to analyze the cytokine profiles with the RayBiotech^®^ Human Cytokine Antibody Array C series 4000 (RayBiotech, Norcross, GA, USA) following the manufacturers’ instructions. A total of 291 cytokines were evaluated as listed in Supplementary Table 1. The detection was carried out with Aksomics Inc. (Shanghai, CN). Briefly, HOK and SCC15 cells were cultured in a serum-free medium, and supernatants of the cell cultures were collected after 24 h. The array slides were blocked with a blocking buffer, antibody arrays were pretreated with culture supernatant overnight at 4 °C, in triplicate. The slides were then extensively washed and incubated with biotin-conjugated primary antibodies for 2 h. After adequate washing, the slides were incubated with streptavidin-conjugated secondary antibodies for 1 h. Finally, data were normalized to internal positive and negative controls and demonstrated in Supplementary Table 1.

### Enzyme-linked immunosorbent assay (ELISA)

CCL2 levels were detected in the cell culture supernatant for 48 h in respective conditions and quantified using Human CCL2 ELISA Kit (Telenbiotech, Guangzhou, GD, CN) according to the manufacturer’s instructions. CCL17 and CCL22 levels were detected in the cell culture supernatant collected from HSC6 and SCC15 cells cultured with or without CCL2 (100 ng/mL) for 24 h and analyzed with Human CCL17 and CCL22 ELISA Kit (Telenbiotech, Guangzhou, GD, CN) according to the manufacturer’s instructions, respectively. Briefly, 50 uL of standard or sample was added to each well, followed by the addition of 100 μL of enzyme conjugate to standard wells and sample wells except the blank well, and incubated for 60 min at 37 °C. The microtiter plates were washed five times and then incubated with freshly mixed substrate solution for 15 min at 37 °C in the dark, and the reaction was stopped by adding 2 N H_2_SO_4_. The absorbance was measured at 450 nm with a microplate reader. The concentration of CCL2, CCL17, or CCL22 was calculated with a standard curve obtained using the absorbance value.

### Human tissue microarrays

Human tissue microarrays OR208 were selected and purchased from Alenabio (Xi’an, SN, CN) for the research. Each tissue microarray contains 60 cases of human oral squamous cell carcinoma tissues and nine cases of normal oral epithelial tissues (each case took three samples and a few samples lacked epithelial components). Details of the human tissue microarrays containing patient samples were shown in Supplementary Table 2.

### Immunohistochemistry staining (IHC)

For IHC, the tissue sections or tissue microarrays were deparaffinized in xylene twice and rehydrated in a graded series of ethanol (100, 95, 85, and 75% ethanol) and phosphate-buffered saline (PBS, pH 7.4) three times. Antigen retrieval was performed by heating the tissue sections at 60 °C in 0.01 M sodium citrate buffer (pH 6.0) in a microwave oven for 20 min and naturally cooled to room temperature. The endogenous peroxidase activity was blocked by 3% hydrogen peroxide for 10 min. After rinsing three times with PBS, tissue sections or tissue microarrays were incubated with normal goat serum for 10 min at room temperature to prevent nonspecific antibody binding. Subsequently, the sections were incubated with different primary antibodies at 4 °C overnight. Then the sections or tissue microarrays were washed in PBS and incubated with goat anti-rabbit IgG (Dako, Glostrup, Denmark) secondary antibody for 30 min at room temperature. The tissue sections or tissue microarrays were then stained with diaminobenzidine (DAB) as a substrate chromogen and counterstained with hematoxylin. Deltopectoral lymph nodes were used as positive controls, and the negative control was performed by substituting the primary antibody with the primary antibody diluent. Subsequently, the specimens were mounted and observed under a light microscope at 100 and 400 magnifications. The antibodies used in this experiment are shown in Table 1.

**Table 1:** The list of antibodies used in this experiment.

Name	Manufacturer	Product #	Application
**Primary Antibodies**			
Anti-CCL2 (Human)	Abcam	ab9669	1:200 (IHC); 1:1000 (WB)
Anti-CCL2 (Mouse, Rat)	Abcam	ab8101	1:10 (IHC)
Anti-CCR4	Novus	NB100-717	1:100 (IHC); 1:1000 (WB)
Anti-Vav2 (phospho- Y172)	Abcam	ab86695	1:200 (IHC); 1:1000 (WB)
Anti-Phospho-Myosin Light Chain 2 (Thr18/Ser19)	CST	#3674 s	1:200 (IHC); 1:500 (WB)
Anti-Luciferase	Abcam	ab16466	1:200 (IHC)
Anti-β-Actin	CST	#3700 s	1:100 (IF)
Anti- Rac1	Abcam	ab33186	1:50 (IF); 1:1000 (WB)
Anti-Prex1	CST	13168 s	1:1000 (WB)
Anti-ECT2	Abcam	ab86604	1:1000 (WB)
Anti-Vav2	CST	#2848 s	1:20 (IF); 1:1000 (WB)
Anti- GAPDH	CST	#5174 s	1:1000 (WB)
Anti- Myosin Light Chain 2	CST	#3672 s	1:1000 (WB)
Anti- CCR2	Proteintech	16153-1-AP	1:1000 (WB)
**Secondary Antibodies**			
AlexaFluor 488	CST	#4408 s	1:20 (IF)
AlexaFluor 555	CST	#4417 s	1:20 (IF)
Anti-mouse IgG, HRP-linked antibody	CST	#7076	1:3000 (WB)
Anti-rabbit IgG, HRP-linked antibody	CST	#7074	1:3000 (WB)

### Cell migration assay

To detect the cell migration potential, a transwell migration assay (8-μm pore size; Corning, Corning, NY, USA) was performed. Briefly, 5 × 10^4^ cells/well of HSC6 cells and 1.2 × 10^5^ cells/well of SCC15 cells were resuspended in 200 uL of serum-free media and subsequently seeded into the upper chamber (transwell chambers), and 700 uL of medium supplemented with 10% FBS was added into the lower chamber as a chemoattractant. After 24 h of incubation, cells remaining in the upper inserts were removed carefully, and migrated cells were fixed in 4% paraformaldehyde for 10 min, stained with 0.5% crystal violet (Beyotime Institute of Biotechnology, Shanghai, China) for 15 min. The upper surface of the membranes was gently wiped and cells migrating the bottom surface of the membrane were quantified under the microscope at 100 magnifications. Experiments were performed in triplicate, and a minimum of three random fields per filter was counted using ImageJ. In addition, wound healing assays were also adopted. HSC6 and SCC15 cells were seeded separately into six-well plates at a density of 5 × 10^5^ cells/well and cultured in a monolayer for 24 h. A 20–200 μL pipette tip. A sterile 200-μL pipette tip was then held vertically to scratch across each well and washed with PBS. Subsequently, adherent cells were cultured in a serum-free medium to allow wound healing. After 0 and 24 h, cellular migration toward the scratched area was monitored and imaged using a phase-contrast microscope at 50 magnifications. The scratch area of three independent experiments was measured by ImageJ.

### Scanning electron microscopy (SEM)

Cells on coverslips were fixed with 2.5% glutaraldehyde overnight at 4 °C. Then, coverslips were dehydrated with a graded series of alcohol (30, 50, 70, 80, 95, and 100% ethanol). After dehydration in alcohol, samples were dried with a critical point drier. The dried samples were viewed under a Hitachi S-3400N (Hitachi, Japan) scanning electron microscope at 3000 magnifications after sputter-coating with gold.

### Immunofluorescence staining

Cells were seeded into observation dishes. After reaching 60–80% confluence, cells were fixed in 4% paraformaldehyde and incubated for 30 min at room temperature. After fixation, the sections were washed with PBS and permeabilized with PBS containing 0.5% TrionX-100 for 15 min at room temperature. Then, cells were blocked with 3% BSA for 30 min at room temperature and subsequently probed with different primary antibodies overnight at 4 °C. Subsequently, the cells were washed in PBS three times and followed by incubation with secondary antibodies for 2 h incubation in the dark at room temperature. The cells were then counterstained with 0.5 ug/mL of DAPI (#4083 s, CST, Danvers, MA, USA) for 5 min at room temperature and washed with PBS three times. The samples were analyzed using a laser scanning confocal microscope at a specific magnification. The antibodies used in this experiment are shown in Table 1.

### Transient transfection of siRNA

To investigate the function of target proteins in HNSCC cells, corresponding small interfering RNA (siRNA) oligonucleotide duplexes (Ribobio, Guangzhou, GD, CN) was used. Using Lipofectamine™ RNAiMAX Transfection Reagent (13778150, Invitrogen, Carlsbad, CA), the siRNA oligonucleotides were transiently transfected into the targeted cells according to the manufacturer’s instructions. For each target gene, we designed three different siRNA sequences and their transfection efficiency was quantified by qRT-PCR and Western blot, respectively (Supplementary Fig. 1). We selected the siRNA with the best inhibition efficiency of the corresponding targeted gene for the following experiment, and the siRNA sequences were presented in Supplementary Table 3.

### RNA extraction and quantitative RT-PCR analysis

Total RNA was extracted from cell samples using TRIzolTM Reagent (15596026, Invitrogen, Carlsbad, CA) according to the manufacturer’s protocol. RNA purity was assessed using the ND-1000 Nanodrop (Nano-Drop Technologies, Wilmington, DE). High-fidelity cDNA was synthesized from purified total RNA using the PrimeScriptTM RT Master Mix (RR036A, TaKaRa, Kusatsu city, JPN). cDNAs were amplified using LightCycler^®^ 480 SYBR Green I Master mix (04707516001, Roche, Basel, CH) on a Roche LightCycler^®^ 96 Instrument according to the manufacturer’s instructions. The amplification reaction included an initial denaturation at 95 °C for 5 min, followed by 45 cycles of denaturation at 95 °C for 10 s, annealing at 60 °C for 20 s, and extension at 72 °C for 30 s, respectively. The expression level of target mRNA was normalized to the internal control GAPDH, using the mean value of the three replicates. The relative gene expression was determined by using the 2^−ΔΔCT^ method. Primers used for qRT-PCR were listed in Supplementary Table 4.

### Lentivirus production and cell transfection

The luciferase fusion plasmids and GFP fusion plasmids were purchased from GeneCopoeiaTM (EX-NEG-Lv217, GeneCopoeiaTM, Rockville, MD). Lentiviral vector production was performed using Lenti-Pac™ HIV Packaging Kit (GeneCopoeiaTM, Rockville, MD) according to the manufacturer’s protocols. HEK-293 cells were transfected with the packaging mixture and a lentiviral or GFP plasmid vector, respectively. Conditioned medium from the transfected HEK-293 cells was used for infecting HNSCC cell lines. At post-transfection, HNSCC cells were incubated in 2.5 ug/mL of puromycin (58-58-2, BioFroxx, Einhausen, GER) for selecting the transfected cells.

### Larval zebrafish transplantation

Adult zebrafish were acquired from Laboratory Animal Center, Sun Yat-sen University, and 15–20 fish were reared in a circulation tank system at 28.5 °C temperature with a 14:10 h light/dark cycle. 0.1 mM 1-phenyl-2-thiourea (PTU) was used to inhibit melanogenesis and generate transparent zebrafish. At 48 h post-fertilization, ~100 GFP-labeled HNSCC cells resuspended in 10 nL of cell medium without serum and were directly transplanted into the circulation via the Duct of Cuvier. The fish were imaged immediately after transplantation, and any fish that did not receive the proper number of cells was discarded. Fish were also imaged at 3 days post-transplant.

### Active GTPase pulldown

To detect GTPase activity, levels of active GTP-bound Rac1 were assessed by an Active Rac1 Pull-Down and Detection Kit (16118, Thermo Fisher Scientific, Waltham, MA, USA). Briefly, sample lysed by GST lysis buffer mixed with 1% protease inhibitor cocktail, and cell lysates which contained equal amounts of protein were exposed to GST-human Pak1-PBD, which contains the binding domain of the Rac1 effector PAK1 overnight. GDP and GTPγ loaded lysates were used as negative and positive active GTPase pulldown controls, respectively. Pulldown samples were resolved by 2X SDS sample buffer and detected by western blot for Rac1. Levels of each GTP-bound Rac1 were normalized to their total protein and compared with each other.

### Western blot

Cells were harvested, and the total protein was extracted with and lysed in RIPA buffer (P0013B, Beyotime, Shanghai, CN) supplemented with a 1% protease inhibitor cocktail (CW2200s, CWbio, Beijing, CN) and 1% phosphatase inhibitor cocktail (CW2383S, CWbio, Beijing, CN) on ice for 30 min. Protein concentration was quantified using BCA Protein Assay Kit (CW0014, CWbio, Beijing, CN) according to the manufacturer’s protocol. Equal amounts of protein were separated on 10% sodium dodecyl sulfate-polyacrylamide gel electrophoresis (SDS-PAGE), and then the proteins were electrophoretically transferred onto polyvinylidene difluoride (PVDF) membranes with 0.22 um pore (Millipore, MA, USA). About 5% BSA was used to block heterogenetic antigen on the membranes for 1 h at room temperature. Subsequently, the membrane was incubated with a specific primary antibody overnight at 4 °C, followed by incubation with the corresponding HRP-conjugated secondary antibodies for 1 h at room temperature. The target bands were visualized using enhanced chemiluminescence (ECL) detection system (Millipore, MA, USA). The antibodies used in this experiment are shown in Table 1.

### Immunoprecipitation (IP)

Cells were lysed in IP lysis buffer, NP-40 lysis buffer (P0013F, Beyotime, Shanghai, CN) supplemented with 1% protease inhibitor cocktail and 1% phosphatase inhibitor cocktail. Cell samples were washed with ice-cold PBS three times and lysed in IP lysis buffer for 30 min on ice. Samples were then centrifuged at 15,000 × *g* for 15 min at 4 °C. The supernatant was collected and then coupled with the corresponding primary antibody overnight at 4 °C. Protein A/G Magnetic Beads (HY-K0202, MedChemExpress, Monmouth Junction, NJ, USA) were added and further incubated for 2 h at 4 °C. Subsequently, the immunoprecipitated beads were washed three times with cold PBS and boiled for 10 min in 2X SDS loading buffer and was subjected to Western blot assay.

### Mouse model

The mouse models used in this study were BALB/c nude mouse lines. Mice were cared for in accordance with the Regulations of Guangdong Province on the Administration of Experimental Animals. BALB/c nude mice were acquired from Laboratory Animal Center, Sun Yat-sen University. Mice were housed under specific pathogen-free conditions with a 12-h light/dark cycle and ad libitum access to tap water and food. We transduced SCC15 cells with lentiviral-luciferase plasmid and selectively expanded the positive stable cells. Each nude mouse was injected with 2.5 million SCC15 cells labeled with luciferase under the skin of the axillary for subcutaneous tumor formation. After tumor implantation, the mice were randomly divided into four groups, including normal saline (NS), CCL2 neutralizing antibody, CCR4 antagonist (Mogamulizumab), and combination group (CCL2 neutralizing antibody combined with CCR4 antagonist), intraperitoneal injection every 3 days. We monitored tumor progression in live animals using in vivo imaging system every week during the treatment and withdrawal period.

### In vivo imaging system

Xenograft tumors were generated by subcutaneous injection of HNSCC cells transfected with luciferase. Subsequently, mice underwent in vivo imaging using the in vivo imaging system (IVIS) spectrum (PerkinElmer) with Living Image software (version 4.4). For bioluminescence studies, animals were injected with an intraperitoneal injection of 200 uL of d-Luciferin (Promega, 1 g dissolved in 66.667 mL DPBS) and anesthesia was induced with 3% isoflurane (RWD) in O_2_ at a flow rate of 2 L/min 8–10 min. Then, luciferase-positive regions were captured and the signal intensity was evaluated at indicated time points post-injection. We imaged mice once a week and monitored them until 3 weeks after the withdrawal of the drug.

### Flow cytometry

The effect of CCL2 and CCR4 antagonist (Mogamulizumab) on the cell cycle of HNSCC cells was monitored using flow cytometry. Briefly, HSC6 and SCC15 cells were seeded in 6-well plates at a cell density of 5000 cells/well. HNSCC cells were cultured with CCL2 (100 ng/ml) combined with or without CCR4 monoclonal antibody (1 ug/ml) and compared with a nontreated group. After 48 h, the cell cycle of HSC6 and SCC15 cells were analyzed with the cell cycle detection kit (CCS012, MULTI SCIENCES). SCC15 and HSC6 cells were incubated with DNA staining solution and Permeabilization kit for 30 min, according to the manufacturer’s instructions. The ratio of cells in the G1, S, G2/M phases of each group was analyzed by flow cytometry (Cytoflex).

### Clone formation assay

HSC6 and SCC15 cells were seeded in six-well plates at a density of 500 cells/well and cultured for 14 days under the indicated conditions. After incubation, cells were fixed with 4% paraformaldehyde for 10 min and stained with 0.5% crystal violet solution for 15 min. Colonies with >50 cells were counted.

### CCK8 viability assay

HSC6 and SCC15 cells were seeded at a cell density of 3000 cells/well into 96-well plates and cultured in the corresponding conditions. After incubation, cell proliferation was analyzed using the Cell Counting Kit-8 (CCK8, Sigma-Aldrich, USA) following the manufacturer’s protocol. We added 10% CCK8 solution (C0037, Beyotime) to each well for 0.5 h. The absorbance was measured at 450 nm using a microplate reader.

### Statistical analysis

Normally distributed data were presented as mean ± SD. Comparisons among three or more groups were assessed using a one-way analysis of variance (ANOVA). A Chi-square test or Fischer’s exact test was used to identify the differences between the two groups. All tests were two-tailed, and *P* < 0.05 was considered statistically significant. All statistical analyses were performed using SPSS 20.0 (SPSS, Chicago, IL, USA).

## Supplementary information

Figure-1 Original WB

Figure-3C&3D Original WB

Figure-3G&3H Original WB

Figure-4A Original WB

Figure-4C&4D Original WB

Figure-4E Original WB

Supplementary figure-1 Original WB

Supplementary figure-3 Original WB

Supplementary figure-4 Original WB

Supplementary figure-6 Original WB

Supplementary figure-7 Original WB

aj-checklist CDDIS-21-3588

Supplementary Table 1

Supplementary Table 2

Supplementary Table 3

Supplementary Table 4

Supplementary figure legends

Supplement figure-1

Supplement figure-2

Supplement figure-3

Supplement figure-4

Supplement figure-5

Supplement figure-6

Supplement figure-7

Supplement figure-8

## Data Availability

The datasets used and/or analyzed during the current study are available from the corresponding author on reasonable request.
