# Supplementary figures and images for: Targeting CCL2-CCR4 axis suppress cell migration of head and neck squamous cell carcinoma
Source: Cell Death Dis. 2022 Feb 17;13(2):158. doi: 10.1038/s41419-022-04610-5 (PMC8854715; doi:10.1038/s41419-022-04610-5)

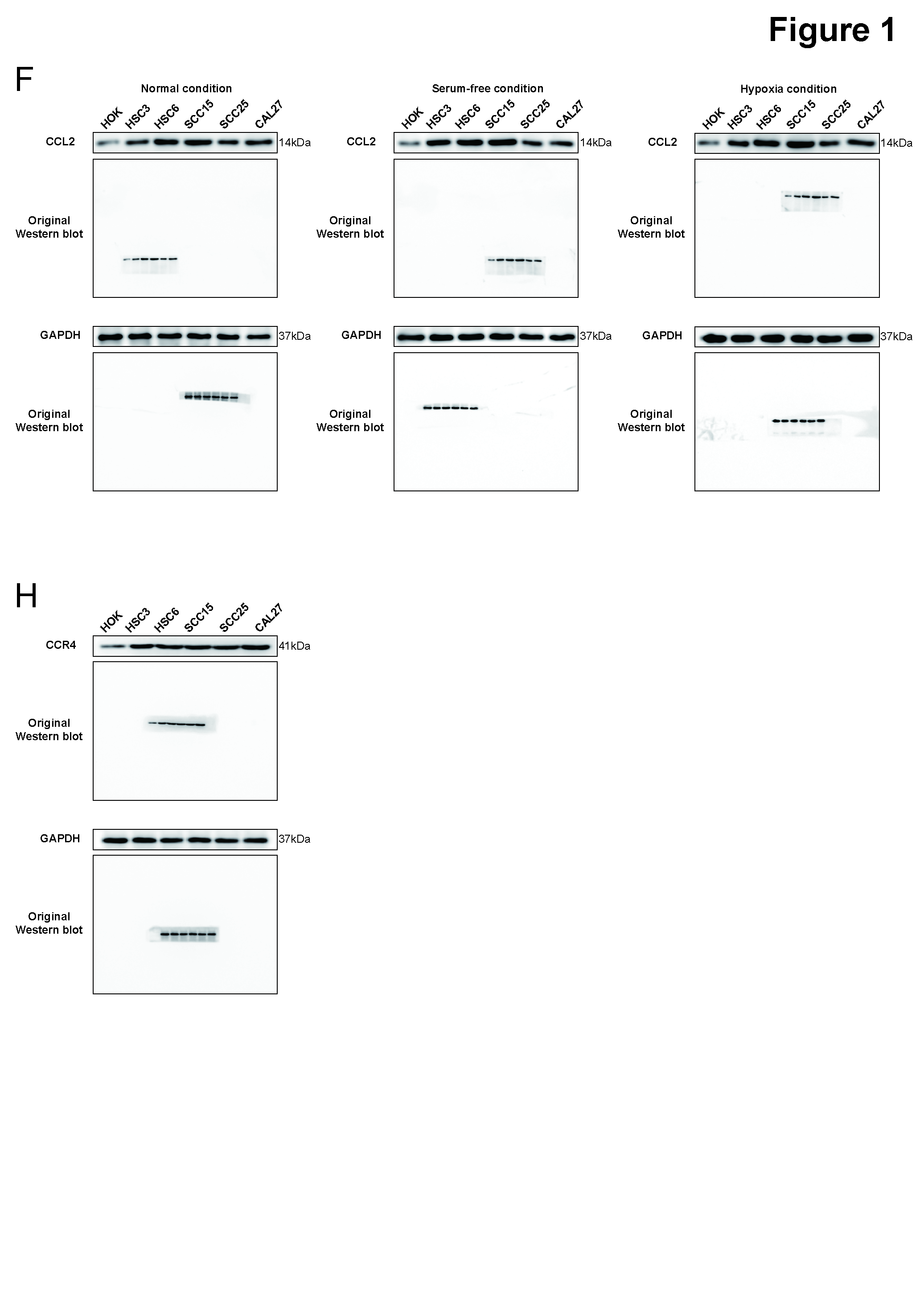

Supplement: Supplementary file 1 — Figure-1 Original WB [file 41419_2022_4610_MOESM1_ESM.tif]

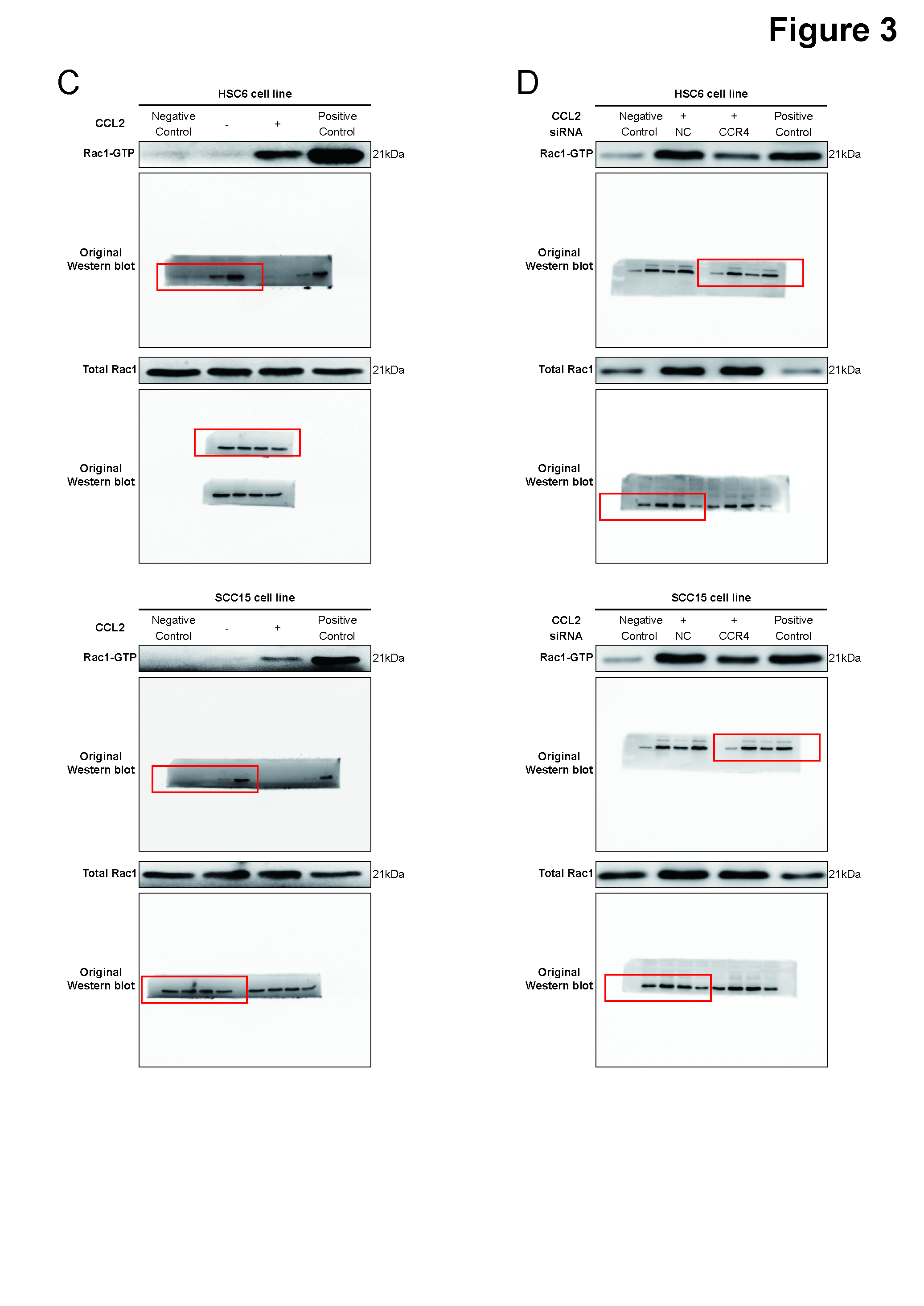

Supplement: Supplementary file 2 — Figure-3C&3D Original WB [file 41419_2022_4610_MOESM2_ESM.tif]

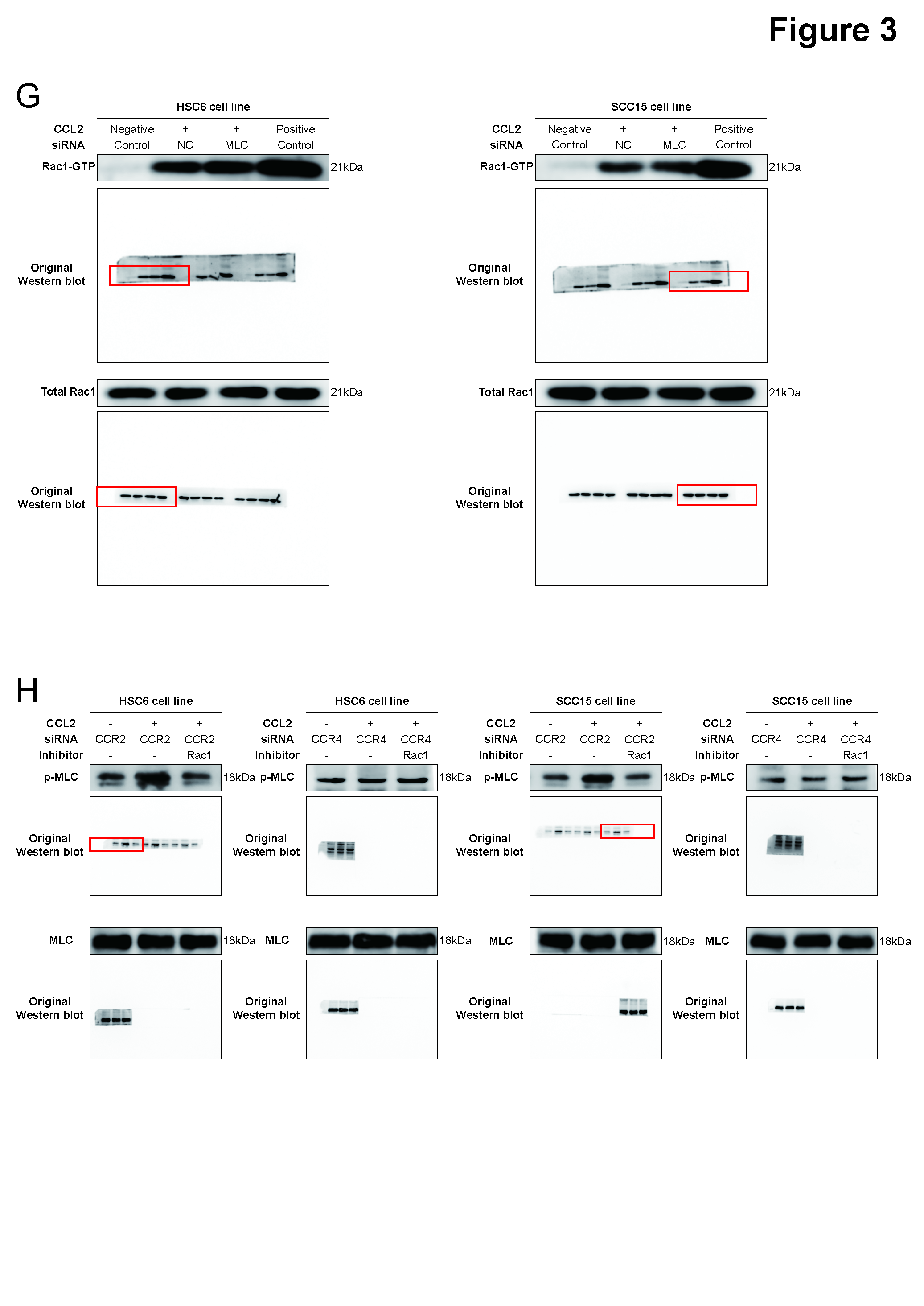

Supplement: Supplementary file 3 — Figure-3G&3H Original WB [file 41419_2022_4610_MOESM3_ESM.tif]

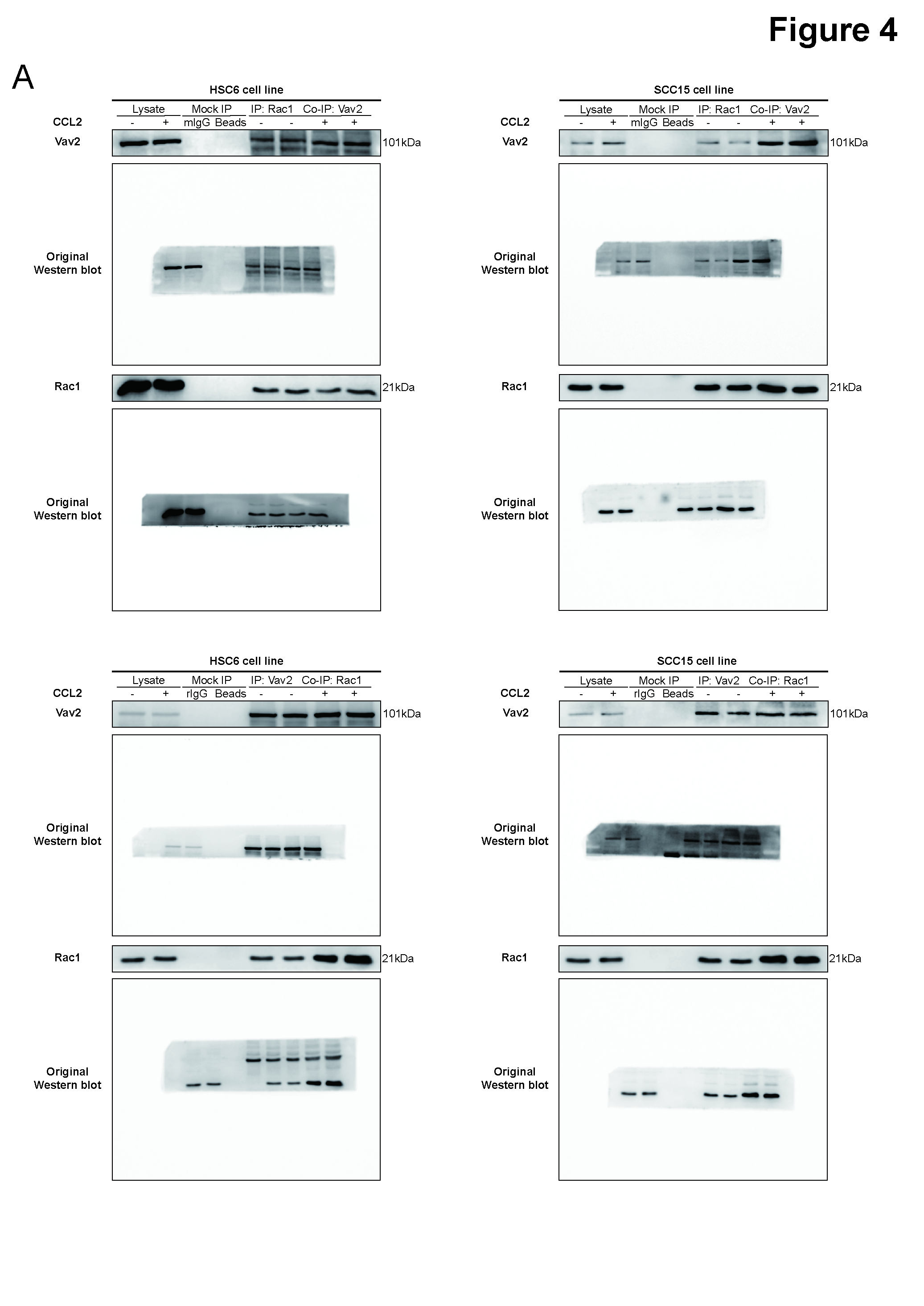

Supplement: Supplementary file 4 — Figure-4A Original WB [file 41419_2022_4610_MOESM4_ESM.tif]

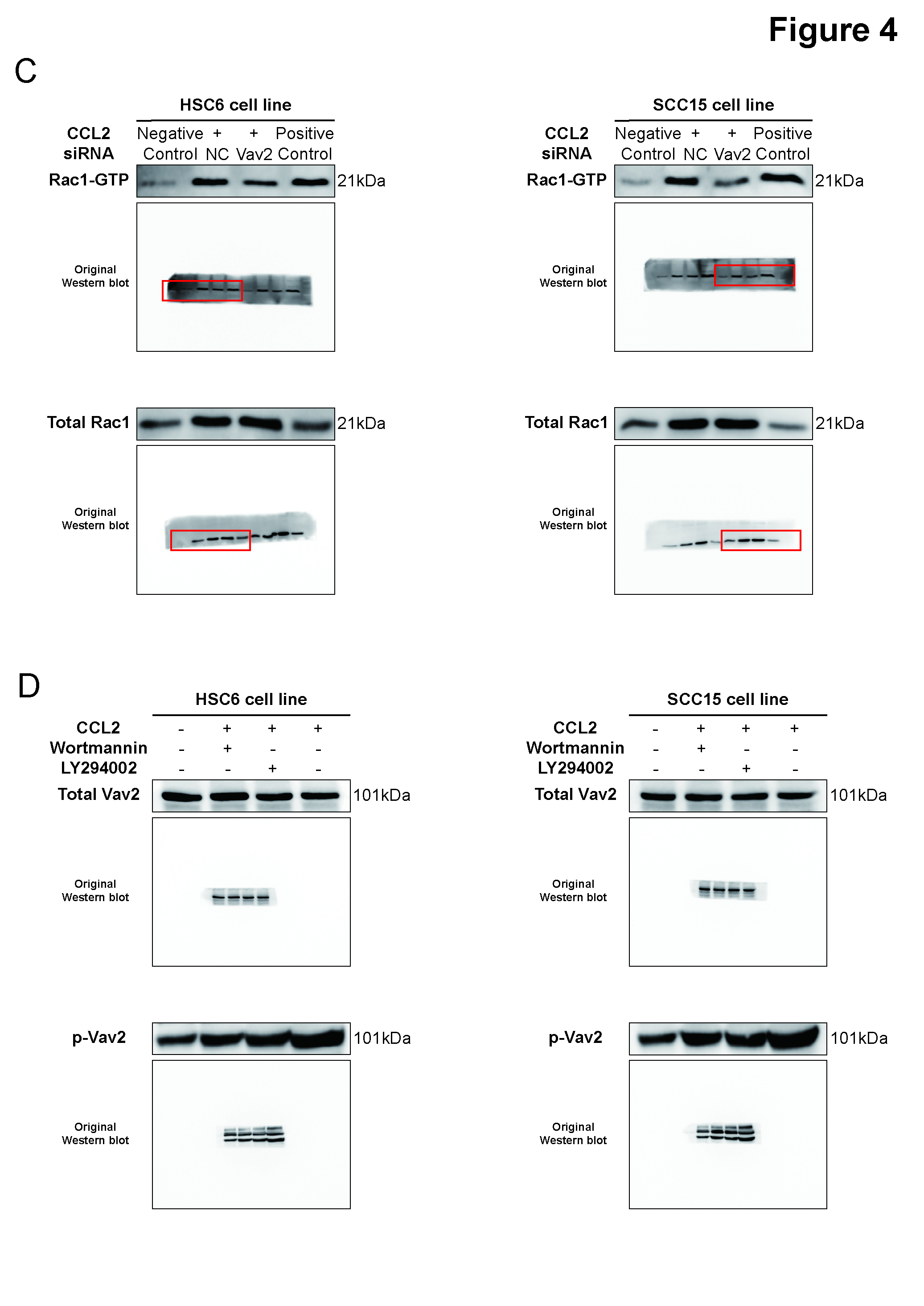

Supplement: Supplementary file 5 — Figure-4C&4D Original WB [file 41419_2022_4610_MOESM5_ESM.tif]

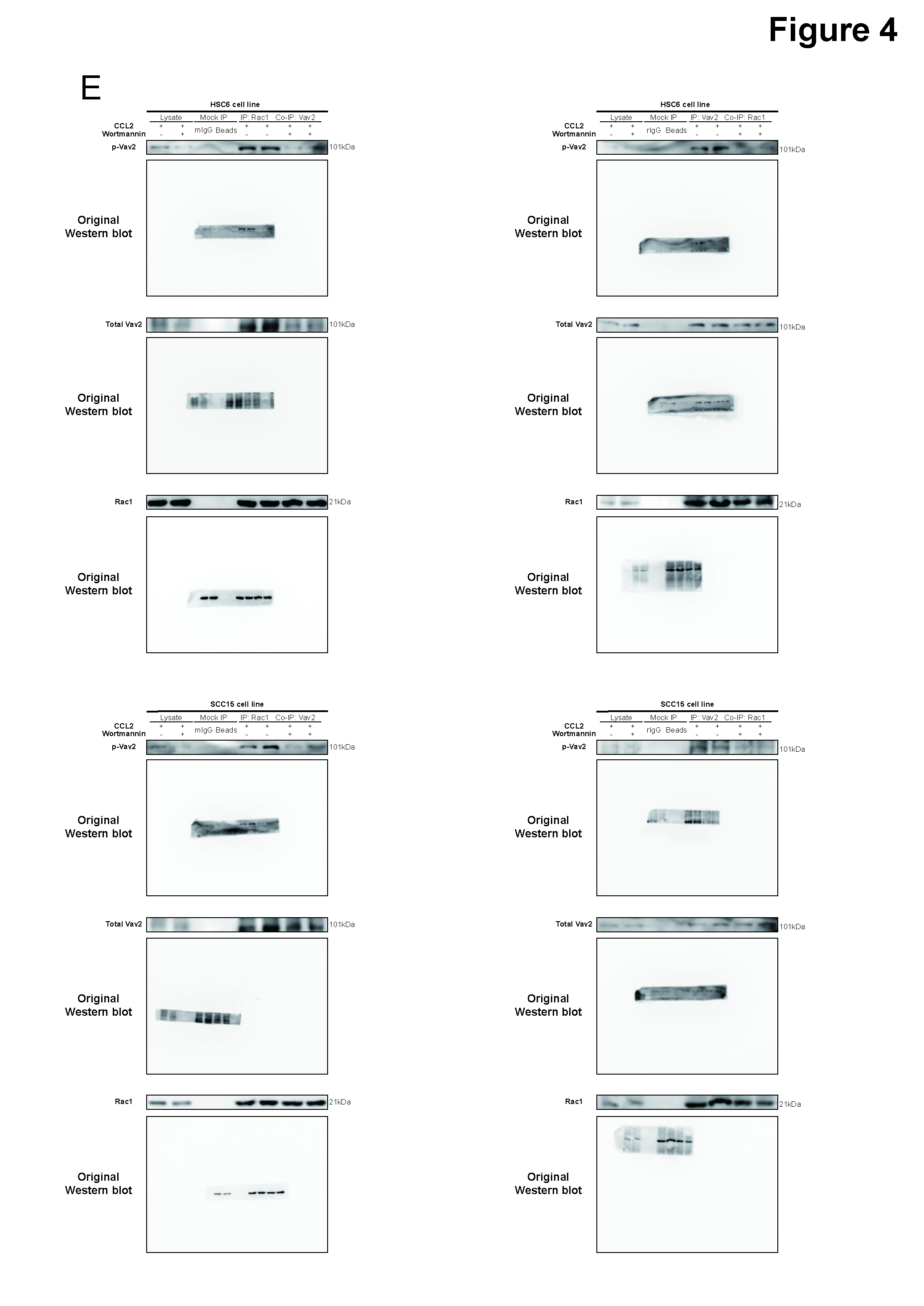

Supplement: Supplementary file 6 — Figure-4E Original WB [file 41419_2022_4610_MOESM6_ESM.tif]

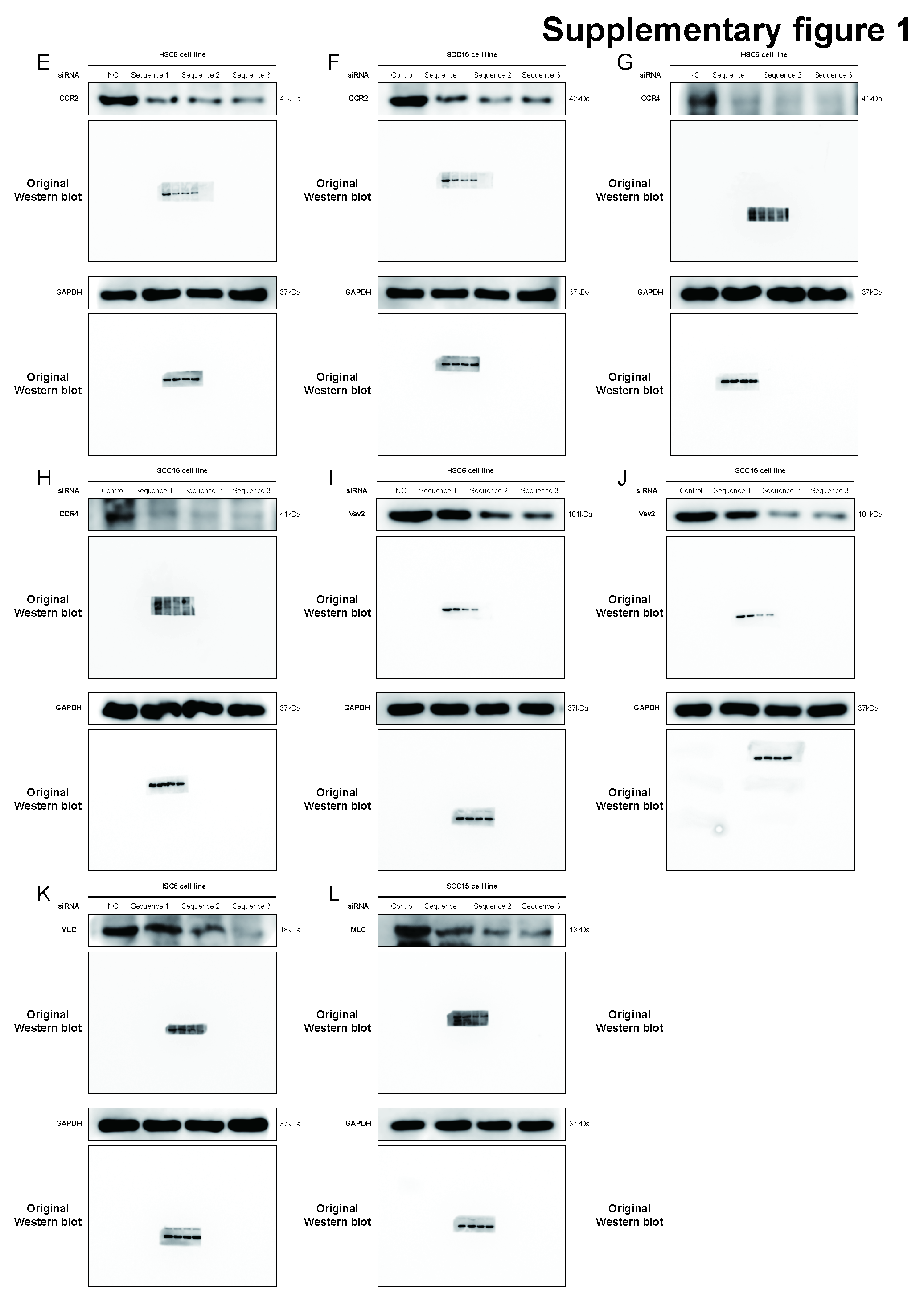

Supplement: Supplementary file 7 — Supplementary figure-1 Original WB [file 41419_2022_4610_MOESM7_ESM.tif]

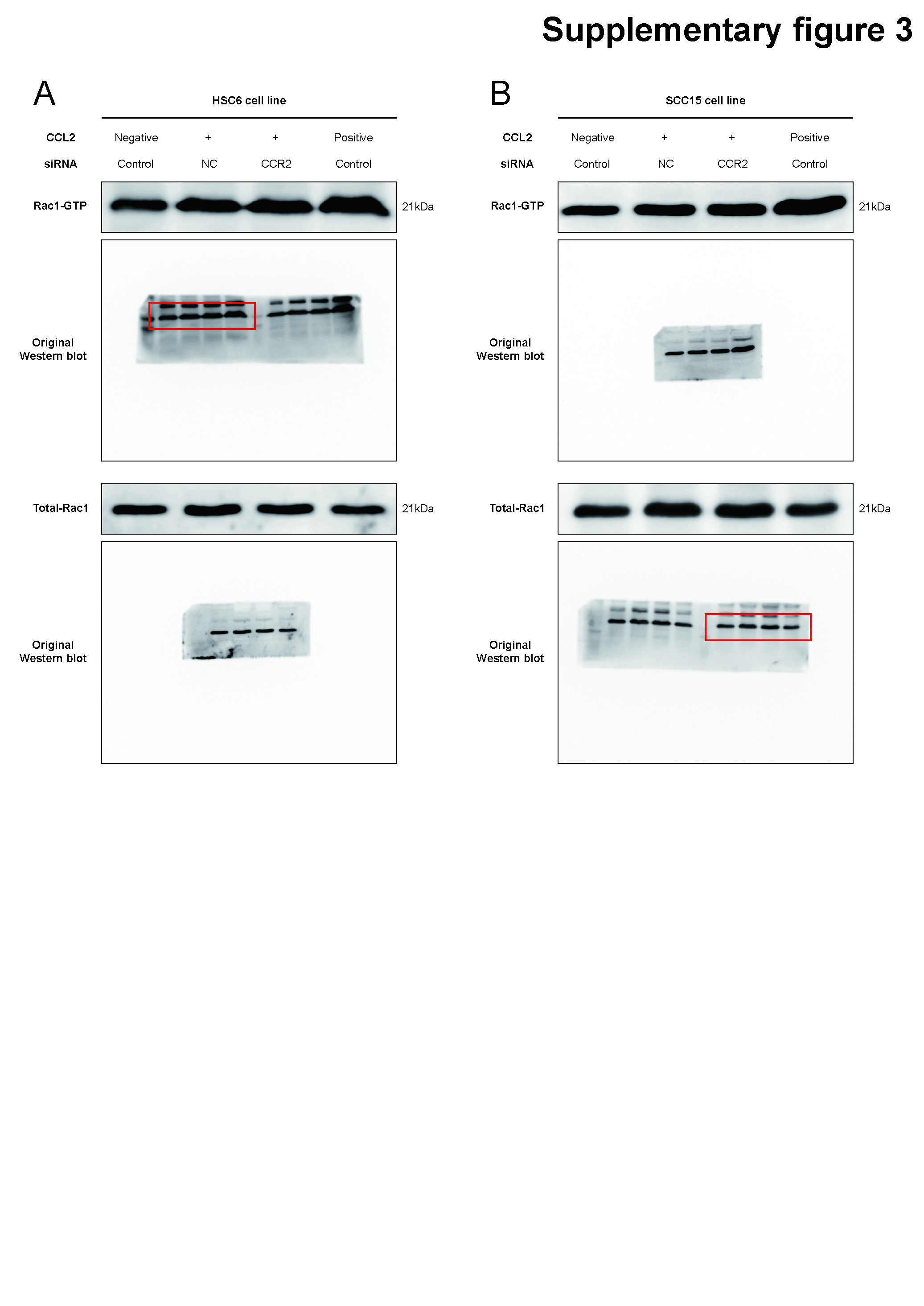

Supplement: Supplementary file 8 — Supplementary figure-3 Original WB [file 41419_2022_4610_MOESM8_ESM.tif]

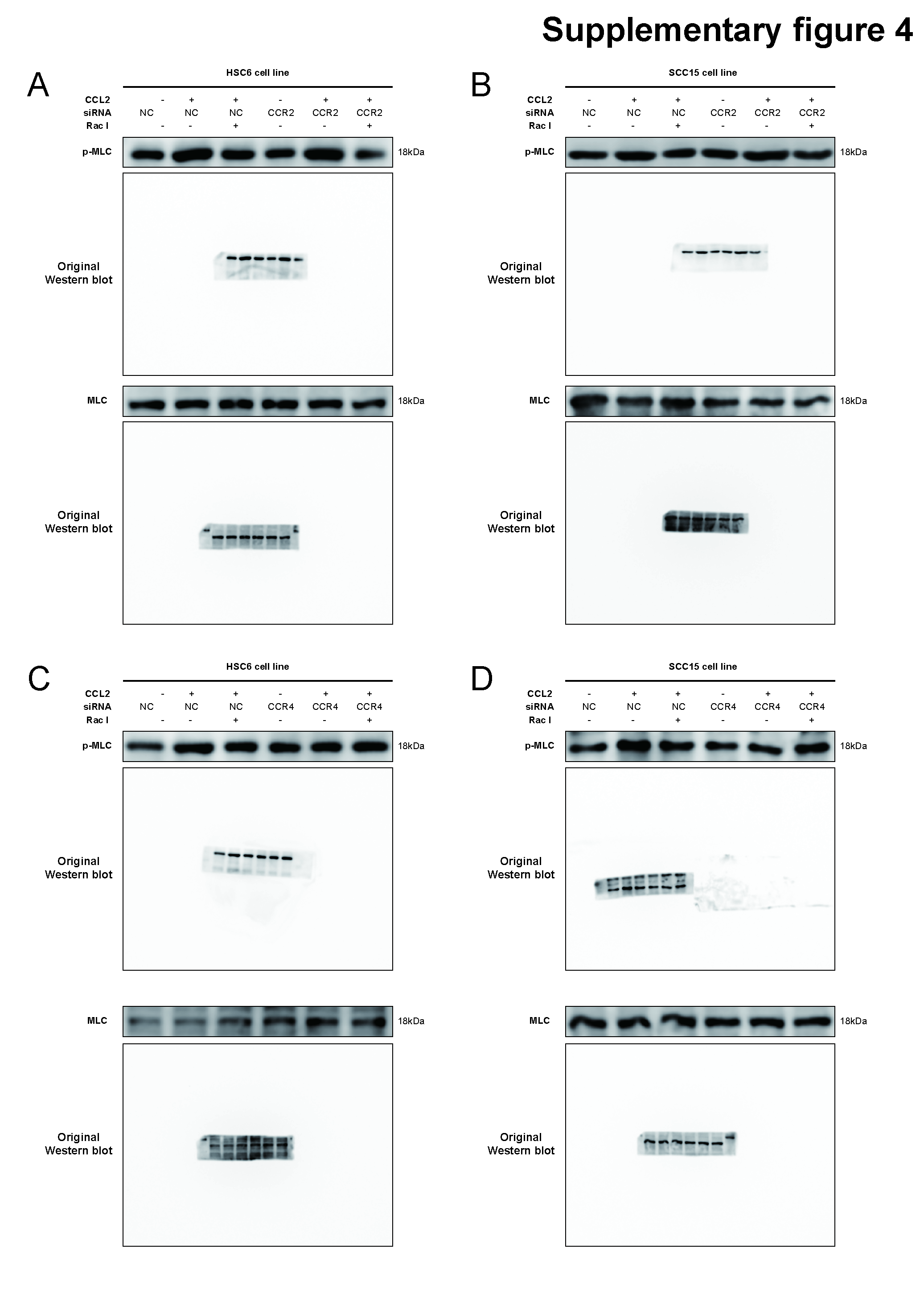

Supplement: Supplementary file 9 — Supplementary figure-4 Original WB [file 41419_2022_4610_MOESM9_ESM.tif]

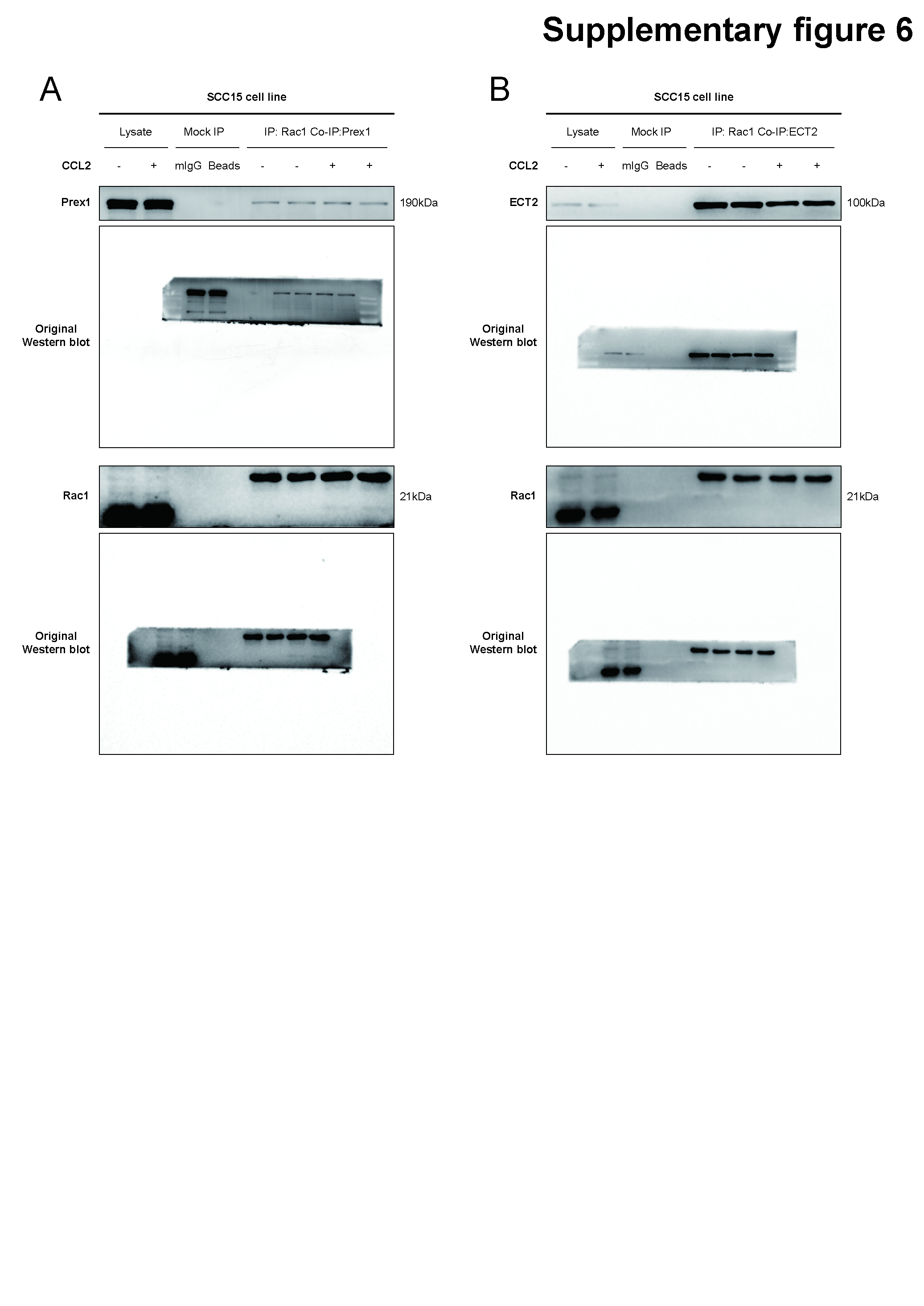

Supplement: Supplementary file 10 — Supplementary figure-6 Original WB [file 41419_2022_4610_MOESM10_ESM.tif]

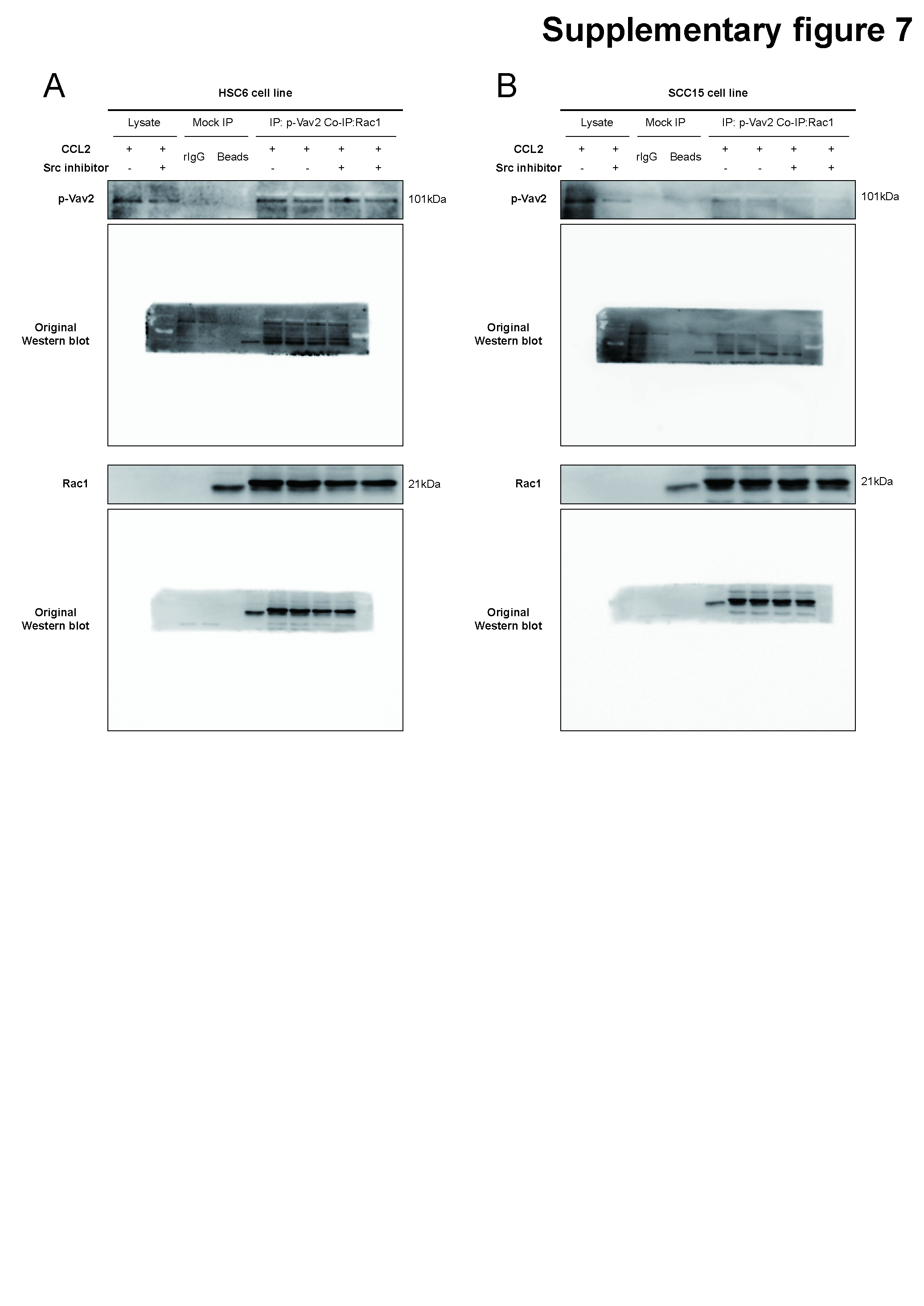

Supplement: Supplementary file 11 — Supplementary figure-7 Original WB [file 41419_2022_4610_MOESM11_ESM.tif]

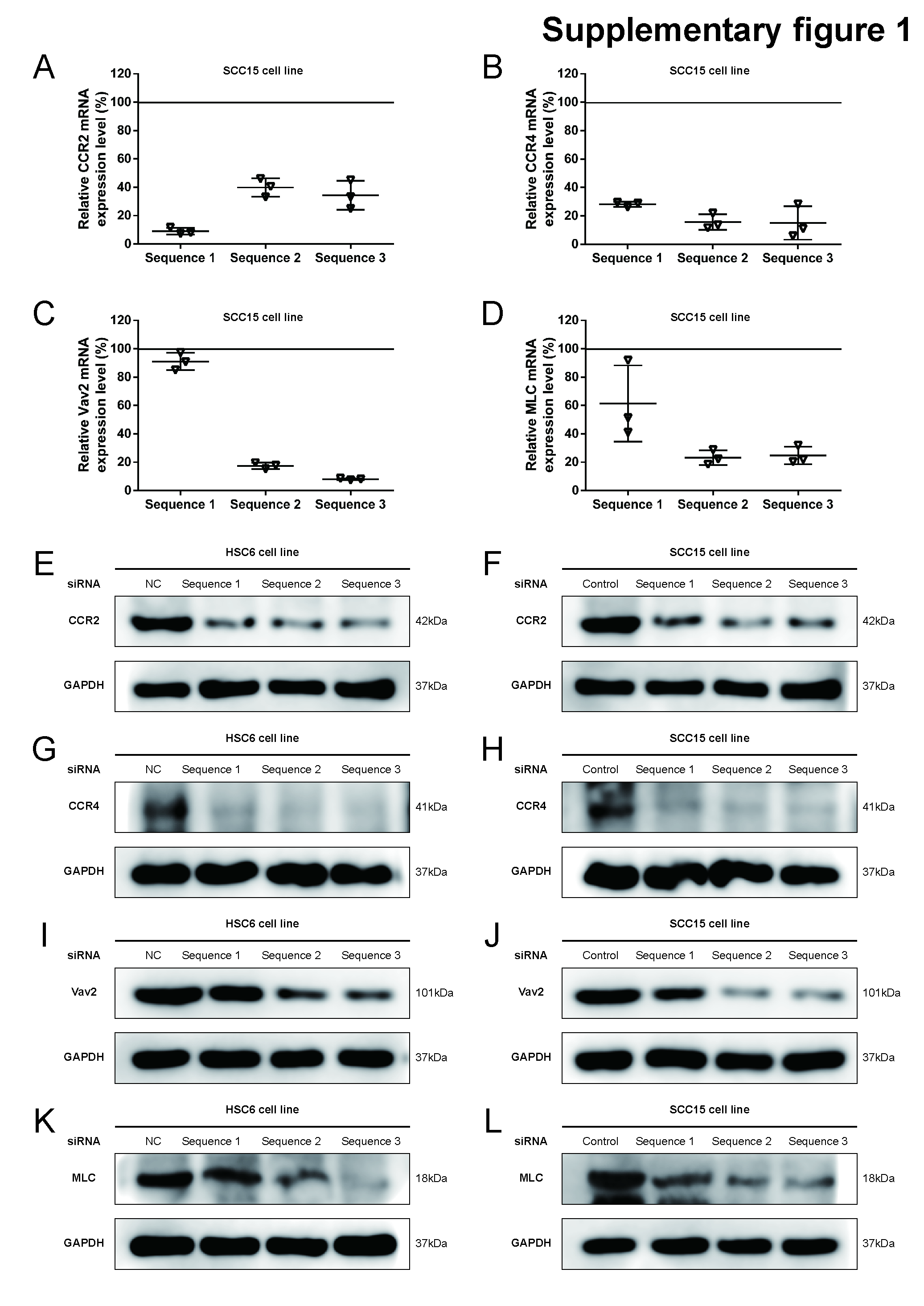

Supplement: Supplementary file 18 — Supplement figure-1 [file 41419_2022_4610_MOESM18_ESM.tif]

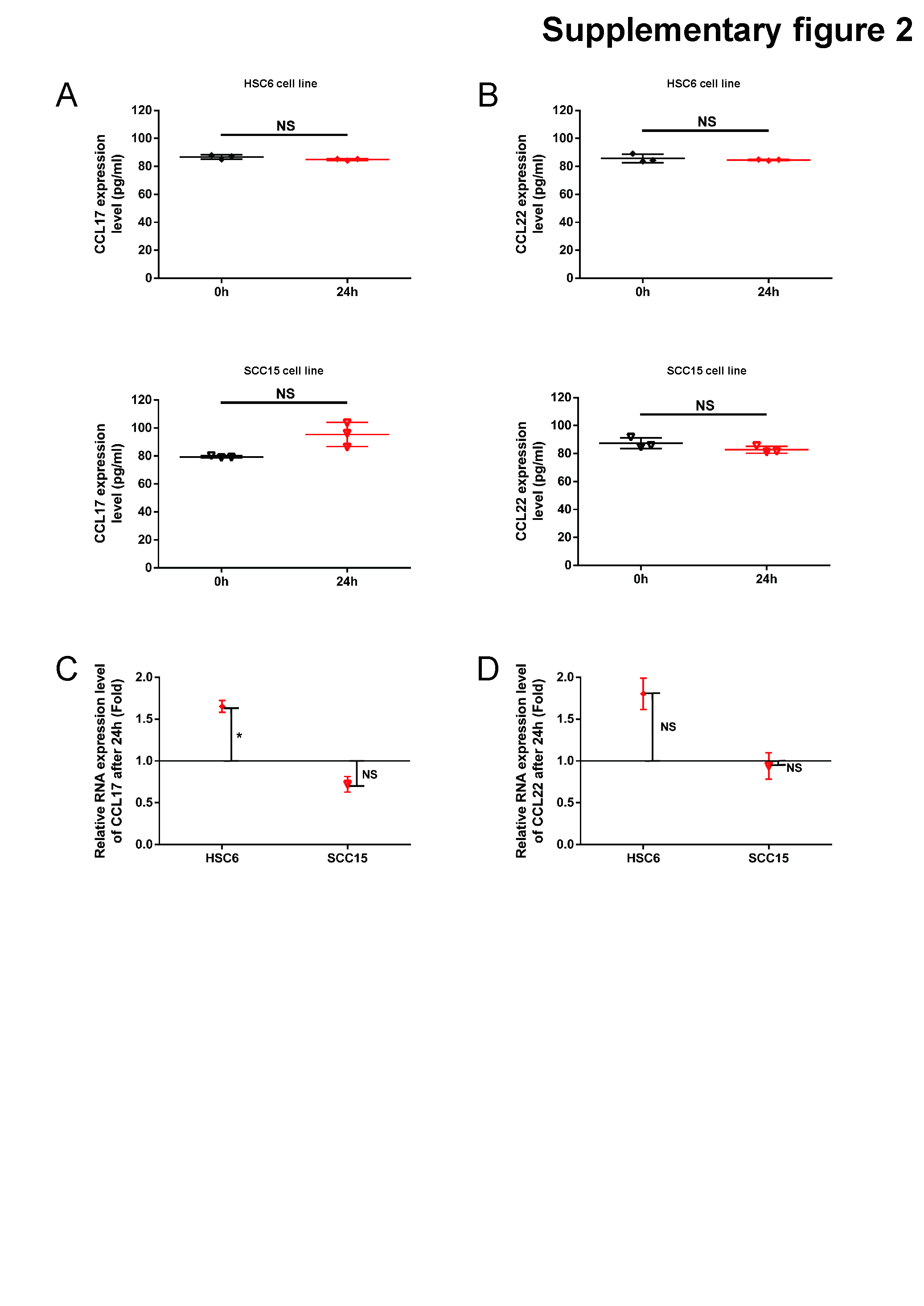

Supplement: Supplementary file 19 — Supplement figure-2 [file 41419_2022_4610_MOESM19_ESM.tif]

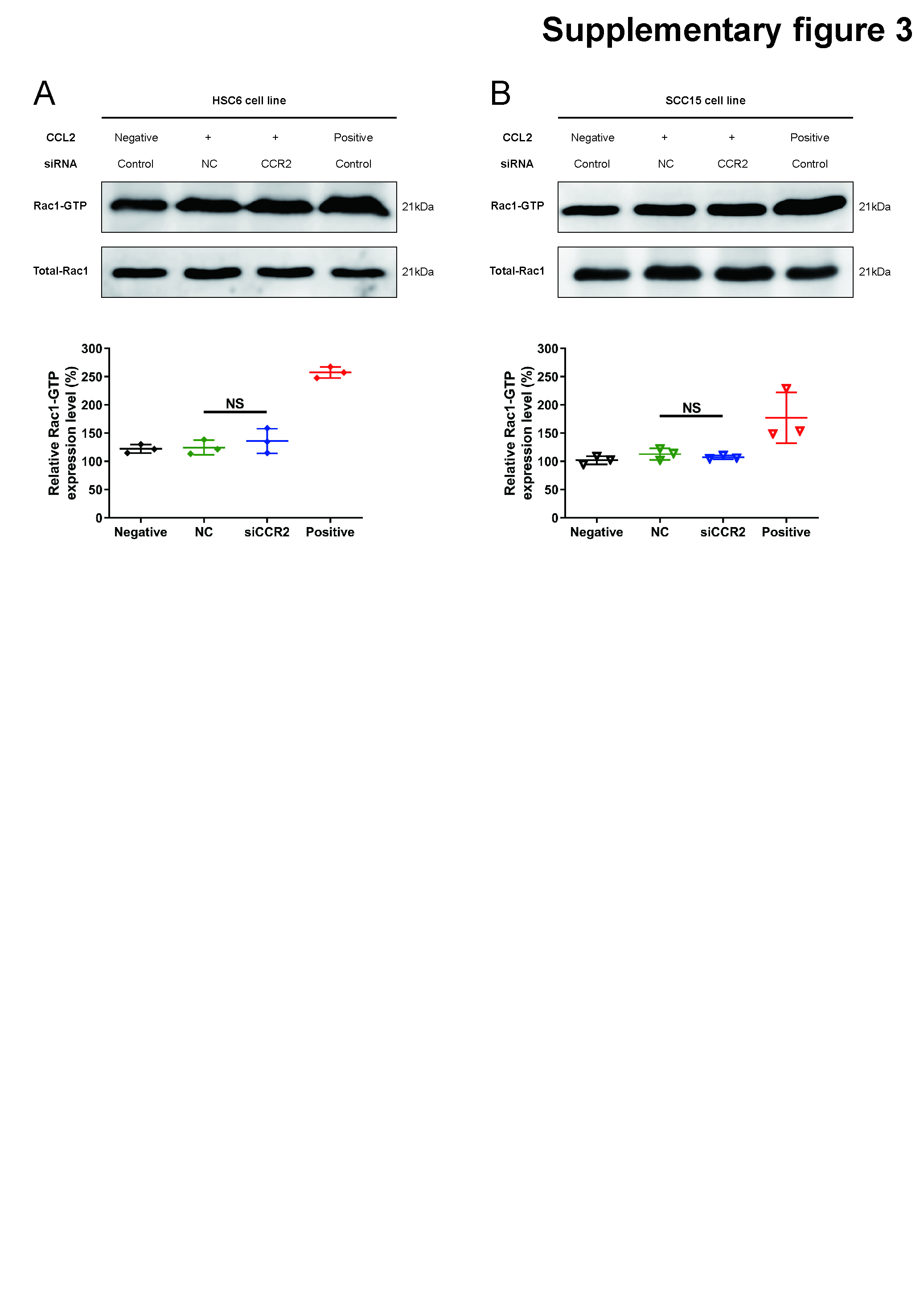

Supplement: Supplementary file 20 — Supplement figure-3 [file 41419_2022_4610_MOESM20_ESM.tif]

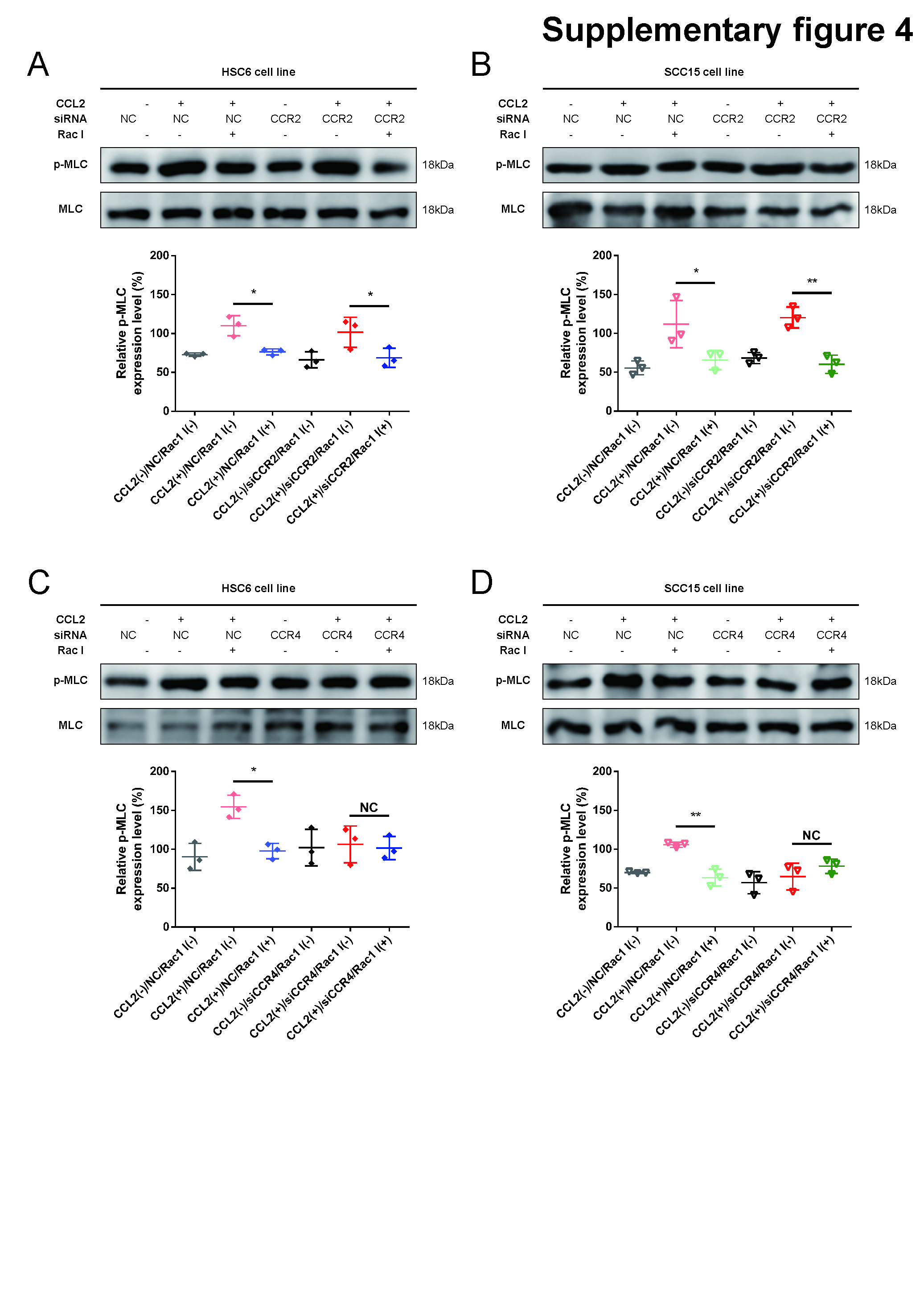

Supplement: Supplementary file 21 — Supplement figure-4 [file 41419_2022_4610_MOESM21_ESM.tif]

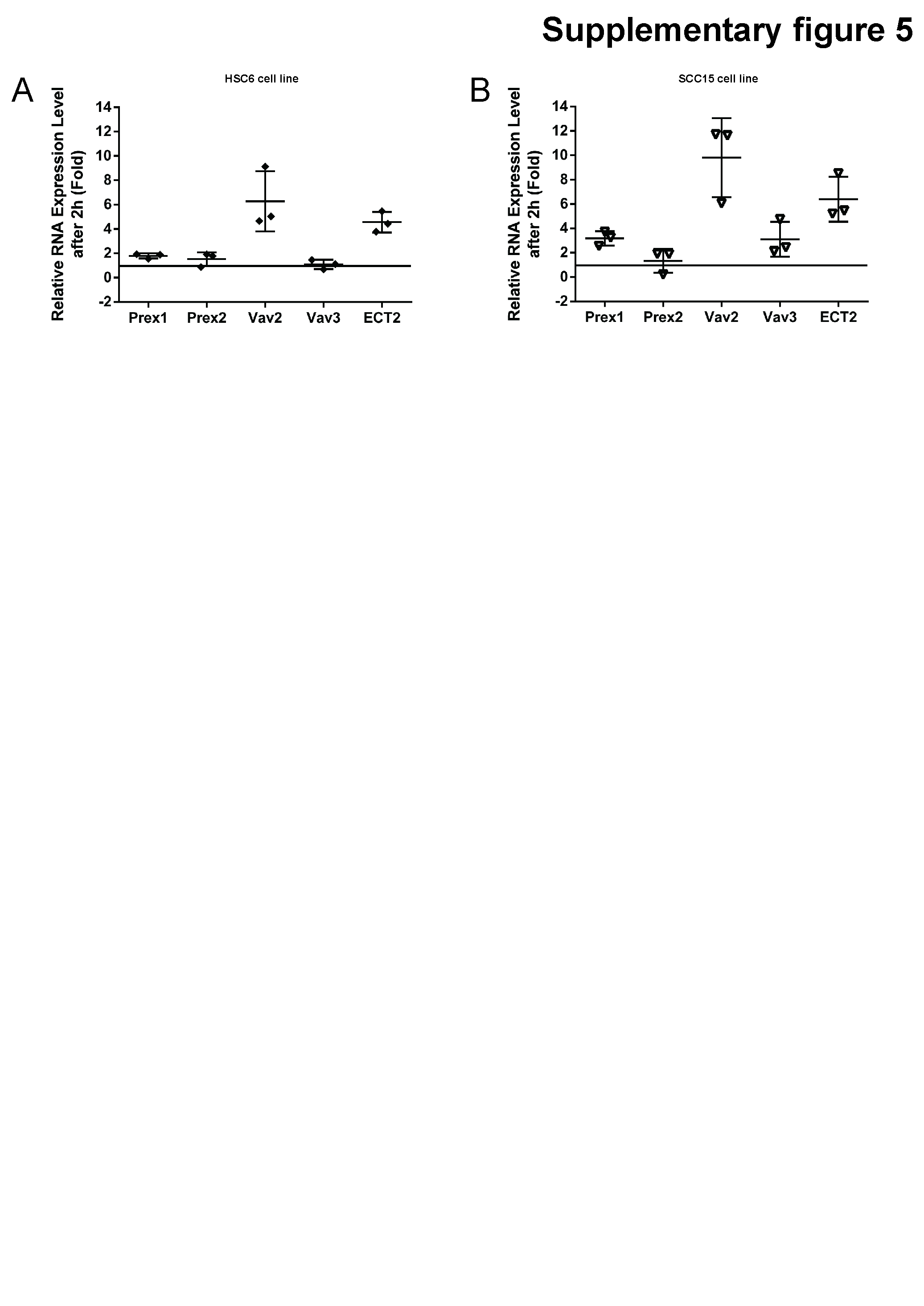

Supplement: Supplementary file 22 — Supplement figure-5 [file 41419_2022_4610_MOESM22_ESM.tif]

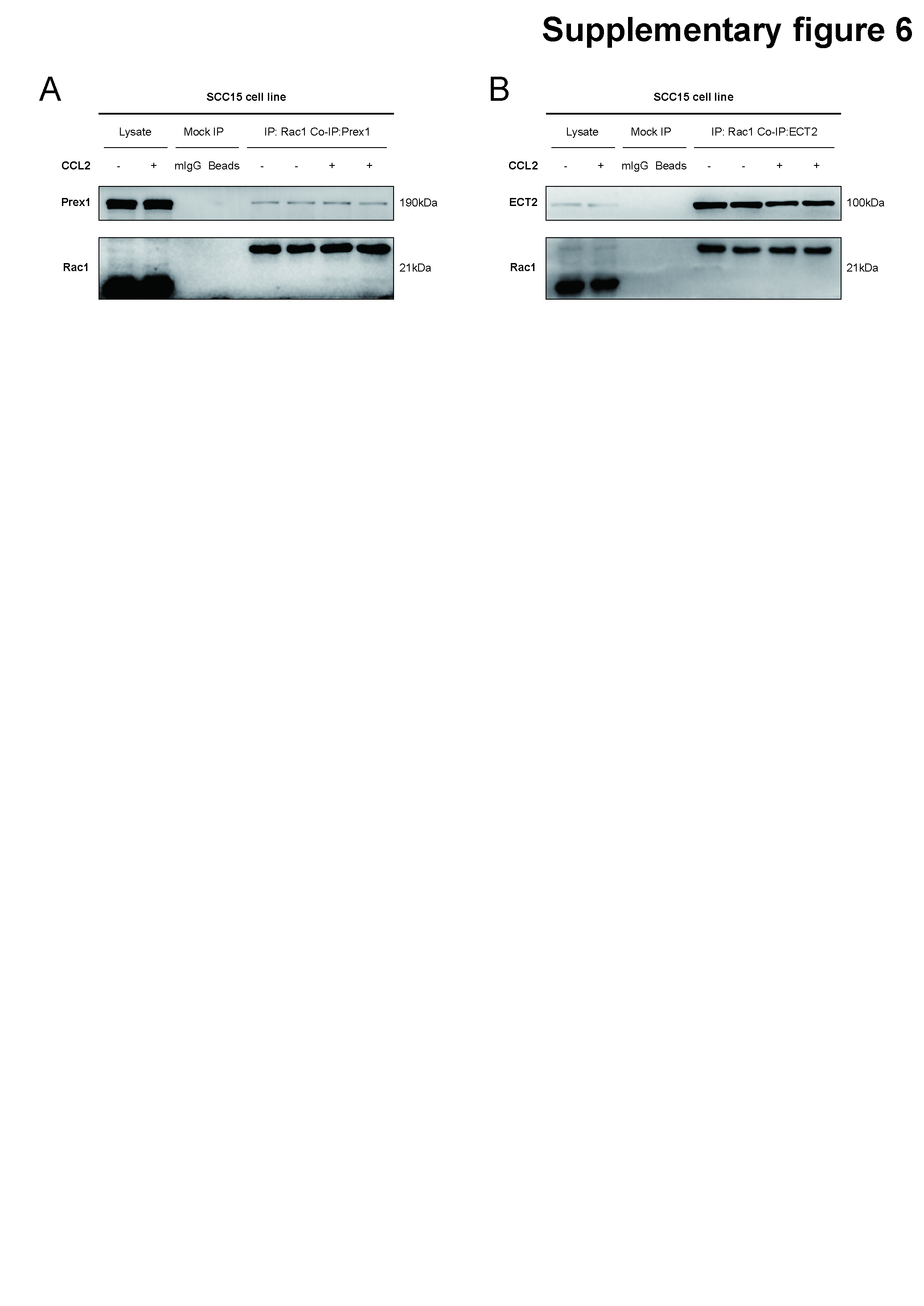

Supplement: Supplementary file 23 — Supplement figure-6 [file 41419_2022_4610_MOESM23_ESM.tif]

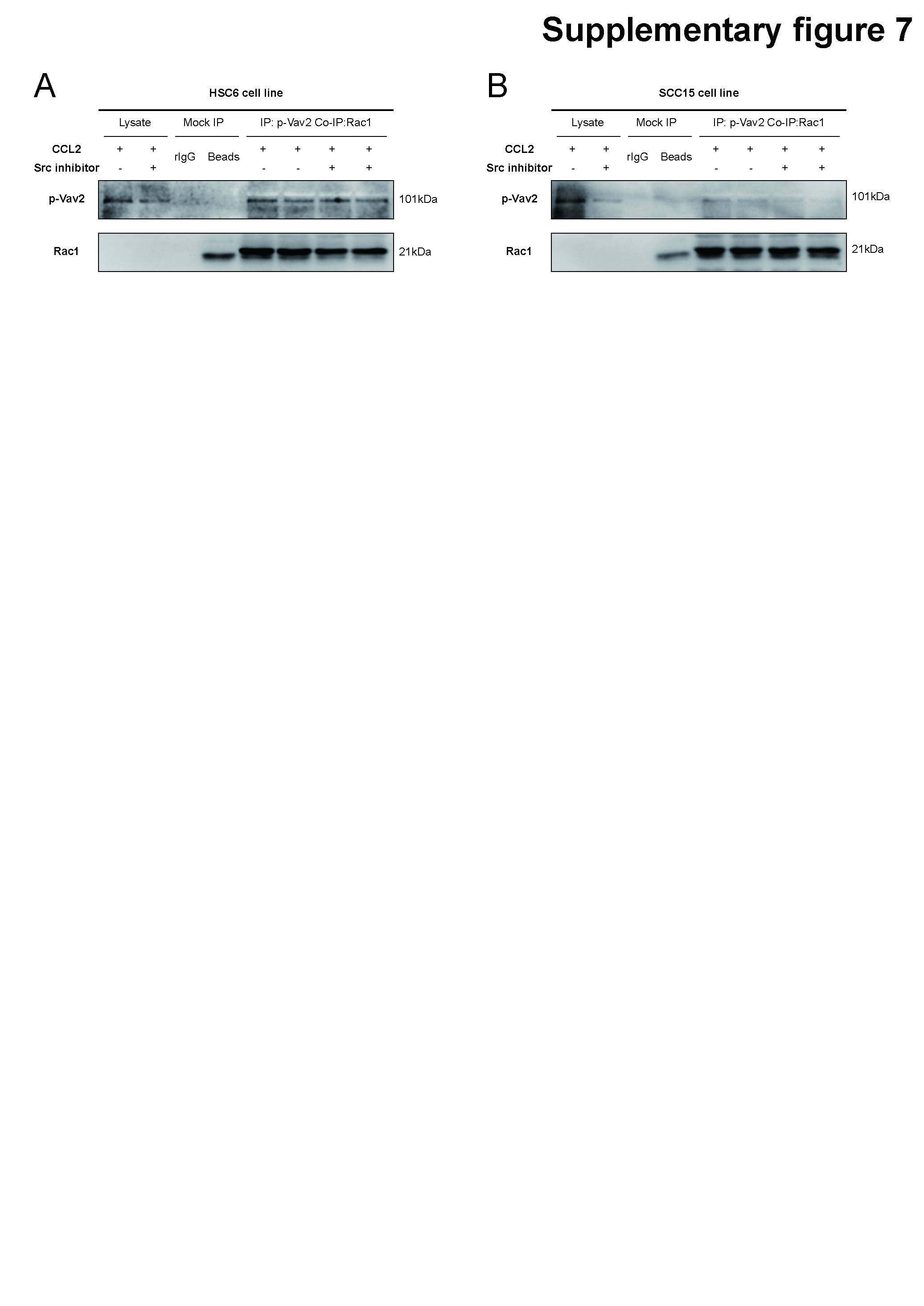

Supplement: Supplementary file 24 — Supplement figure-7 [file 41419_2022_4610_MOESM24_ESM.tif]

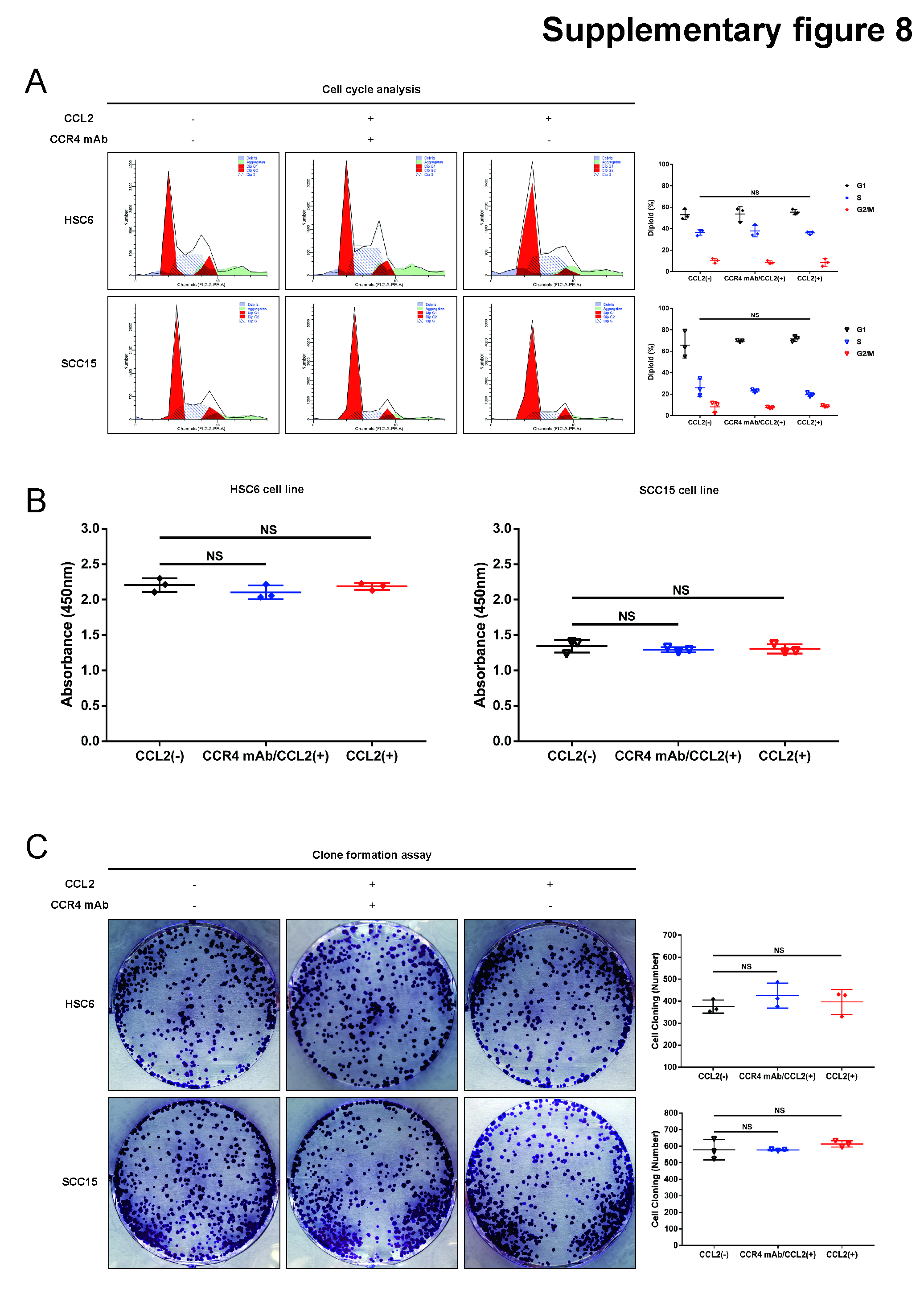

Supplement: Supplementary file 25 — Supplement figure-8 [file 41419_2022_4610_MOESM25_ESM.tif]
