# Supplementary material for: Targeting CCL2-CCR4 axis suppress cell migration of head and neck squamous cell carcinoma
Source: Cell Death Dis. 2022 Feb 17;13(2):158. doi: 10.1038/s41419-022-04610-5 (PMC8854715; doi:10.1038/s41419-022-04610-5)
Supplement: Supplementary file 14 — Supplementary Table 2 [file 41419_2022_4610_MOESM14_ESM.docx]

**Supplementary Table 2. Details of patient samples of OR208**

OR208 contains 60 cases of human oral squamous cell carcinoma tissues and 9 cases of normal oral epithelial tissues (each case took 3 samples and few samples lacked epithelial components).

| **No. Age Sex Organ Pathology diagnosis Grade TNM Stage Tissue Id Type** | | | | | | | | | |
| --- | --- | --- | --- | --- | --- | --- | --- | --- | --- |
| A1 | 64 | M | Oral cavity | Squamous cell carcinoma | 1 | T1N0M0 |  | Doc040556 | malignant |
| A2 | 64 | M | Oral cavity | Squamous cell carcinoma | 1 | T1N0M0 |  | Doc040556 | malignant |
| A3 | 64 | M | Oral cavity | Squamous cell carcinoma | 1 | T1N0M0 |  | Doc040556 | malignant |
| A4 | 42 | M | Oral cavity | Squamous cell carcinoma (necrotic tissue) | - | T1N0M0 |  | Doc031984 | malignant |
| A5 | 42 | M | Oral cavity | Squamous cell carcinoma (sparse) | 1 | T1N0M0 |  | Doc031984 | malignant |
| A6 | 42 | M | Oral cavity | Squamous cell carcinoma | 1 | T1N0M0 |  | Doc031984 | malignant |
| A7 | 59 | M | Oral cavity | Squamous cell carcinoma | 1 | T1N0M0 |  | Doc050206 | malignant |
| A8 | 59 | M | Oral cavity | Squamous cell carcinoma | 1 | T1N0M0 |  | Doc050206 | malignant |
| A9 | 59 | M | Oral cavity | Squamous cell carcinoma | 1 | T1N0M0 |  | Doc050206 | malignant |
| A10 | 67 | M | Oral cavity | Squamous cell carcinoma | 1 | T4N0M0 |  | Doc040554 | malignant |
| A11 | 67 | M | Oral cavity | Squamous cell carcinoma | 1 | T4N0M0 |  | Doc040554 | malignant |
| A12 | 67 | M | Oral cavity | Squamous cell carcinoma | 1 | T4N0M0 |  | Doc040554 | malignant |
| A13 | 50 | M | Oral cavity | Squamous cell carcinoma | 1 | T3N0M0 |  | Doc050175 | malignant |
| A14 | 50 | M | Oral cavity | Squamous cell carcinoma | 1 | T3N0M0 |  | Doc050175 | malignant |
| A15 | 50 | M | Oral cavity | Squamous cell carcinoma | 1 | T3N0M0 |  | Doc050175 | malignant |
| A16 | 75 | F | Oral cavity | Squamous cell carcinoma | 1 | T1N0M0 |  | Doc041807 | malignant |
| B1 | 75 | F | Oral cavity | Squamous cell carcinoma | 1 | T1N0M0 |  | Doc041807 | malignant |
| B2 | 75 | F | Oral cavity | Squamous cell carcinoma | 1 | T1N0M0 |  | Doc041807 | malignant |
| B3 | 61 | M | Oral cavity | Squamous cell carcinoma | 1 | T2N0M0 |  | Doc050018 | malignant |
| B4 | 61 | M | Oral cavity | Squamous cell carcinoma | 1 | T2N0M0 |  | Doc050018 | malignant |
| B5 | 61 | M | Oral cavity | Squamous cell carcinoma | 1 | T2N0M0 |  | Doc050018 | malignant |
| B6 | 62 | M | Oral cavity | Squamous cell carcinoma | 1 | T1N0M0 |  | Doc041650 | malignant |
| B7 | 62 | M | Oral cavity | Squamous cell carcinoma | 1 | T1N0M0 |  | Doc041650 | malignant |
| B8 | 62 | M | Oral cavity | Squamous cell carcinoma | 1 | T1N0M0 |  | Doc041650 | malignant |
| B9 | 54 | M | Oral cavity | Squamous cell carcinoma | 1 | T1N0M0 |  | Doc040411 | malignant |

| B10 | 54 | M | Oral cavity | Squamous cell carcinoma | 1 | T1N0M0 |  | Doc040411 | malignant |
| --- | --- | --- | --- | --- | --- | --- | --- | --- | --- |
| B11 | 54 | M | Oral cavity | Squamous cell carcinoma | 1 | T1N0M0 |  | Doc040411 | malignant |
| B12 | 41 | M | Oral cavity | Squamous cell carcinoma | 1 | T2N0M0 |  | Doc031940 | malignant |
| B13 | 41 | M | Oral cavity | Squamous cell carcinoma | 1 | T2N0M0 |  | Doc031940 | malignant |
| B14 | 41 | M | Oral cavity | Squamous cell carcinoma | 1 | T2N0M0 |  | Doc031940 | malignant |
| B15 | 51 | M | Oral cavity | Squamous cell carcinoma | 1 | T4N0M0 |  | Doc040816 | malignant |
| B16 | 51 | M | Oral cavity | Squamous cell carcinoma | 1 | T4N0M0 |  | Doc040816 | malignant |
| C1 | 51 | M | Oral cavity | Squamous cell carcinoma | 1 | T4N0M0 |  | Doc040816 | malignant |
| C2 | 66 | F | Oral cavity | Squamous cell carcinoma | 2 | T1N0M0 |  | Doc040768 | malignant |
| C3 | 66 | F | Oral cavity | Squamous cell carcinoma | 1 -- 2 | T1N0M0 |  | Doc040768 | malignant |
| C4 | 66 | F | Oral cavity | Squamous cell carcinoma | 1 | T1N0M0 |  | Doc040768 | malignant |
| C5 | 70 | M | Oral cavity | Squamous cell carcinoma | 1 | T1N0M0 |  | Doc041012 | malignant |
| C6 | 70 | M | Oral cavity | Squamous cell carcinoma | 1 | T1N0M0 |  | Doc041012 | malignant |
| C7 | 70 | M | Oral cavity | Squamous cell carcinoma | 1 | T1N0M0 |  | Doc041012 | malignant |
| C8 | 41 | F | Oral cavity | Squamous cell carcinoma | 1 | T4N0M0 |  | Doc041027 | malignant |
| C9 | 41 | F | Oral cavity | Squamous cell carcinoma | 1 | T4N0M0 |  | Doc041027 | malignant |
| C10 | 41 | F | Oral cavity | Squamous cell carcinoma | 1 | T4N0M0 |  | Doc041027 | malignant |
| C11 | 36 | F | Oral cavity | Squamous cell carcinoma | 1 | T4N0M0 |  | Doc041198 | malignant |
| C12 | 36 | F | Oral cavity | Squamous cell carcinoma | 1 | T4N0M0 |  | Doc041198 | malignant |
| C13 | 36 | F | Oral cavity | Squamous cell carcinoma | 1 | T4N0M0 |  | Doc041198 | malignant |
| C14 | 57 | F | Oral cavity | Squamous cell carcinoma | 1 | T2N0M0 |  | Doc041301 | malignant |
| C15 | 57 | F | Oral cavity | Squamous cell carcinoma | 1 | T2N0M0 |  | Doc041301 | malignant |
| C16 | 57 | F | Oral cavity | Squamous cell carcinoma | 1 | T2N0M0 |  | Doc041301 | malignant |
| D1 | 51 | M | Oral cavity | Squamous cell carcinoma | 1 | T1N0M0 |  | Doc060869 | malignant |
| D2 | 51 | M | Oral cavity | Squamous cell carcinoma | 1 | T1N0M0 |  | Doc060869 | malignant |
| D3 | 51 | M | Oral cavity | Squamous cell carcinoma | 1 | T1N0M0 |  | Doc060869 | malignant |
| D4 | 43 | M | Oral cavity | Squamous cell carcinoma (fibrofatty tissue) | - | T4N0M0 |  | Doc060866 | malignant |
| D5 | 43 | M | Oral cavity | Squamous cell carcinoma (fibrofatty tissue) | - | T4N0M0 |  | Doc060866 | malignant |
| D6 | 43 | M | Oral cavity | Squamous cell carcinoma (fibrofatty tissue) | - | T4N0M0 |  | Doc060866 | malignant |
| D7 | 39 | M | Oral cavity | Squamous cell carcinoma | 1 | T4N0M0 |  | Doc060821 | malignant |
| D8 | 39 | M | Oral cavity | Squamous cell carcinoma | 1 | T4N0M0 |  | Doc060821 | malignant |
| D9 | 39 | M | Oral cavity | Squamous cell carcinoma | 1 | T4N0M0 |  | Doc060821 | malignant |
| D10 | 57 | M | Oral cavity | Squamous cell carcinoma | 1 | T2N0M0 |  | Doc060796 | malignant |
| D11 | 57 | M | Oral cavity | Squamous cell carcinoma | 1 | T2N0M0 |  | Doc060796 | malignant |
| D12 | 57 | M | Oral cavity | Squamous cell carcinoma | 1 | T2N0M0 |  | Doc060796 | malignant |
| D13 | 52 | F | Oral cavity | Squamous cell carcinoma | 1 | T1N0M0 |  | Doc060669 | malignant |
| D14 | 52 | F | Oral cavity | Squamous cell carcinoma | 1 | T1N0M1 |  | Doc060669 | malignant |
| D15 | 52 | F | Oral cavity | Squamous cell carcinoma | 1 | T1N0M2 |  | Doc060669 | malignant |
|  |  |  |  |  |  |  |  |  |  |

| D16 | 69 | M | Oral cavity | Squamous cell carcinoma | 1 | T3N0M0 |  | Doc040129 | malignant |
| --- | --- | --- | --- | --- | --- | --- | --- | --- | --- |
| E1 | 69 | M | Oral cavity | Squamous cell carcinoma | 1 | T3N0M0 |  | Doc040129 | malignant |
| E2 | 69 | M | Oral cavity | Squamous cell carcinoma | 1 | T3N0M0 |  | Doc040129 | malignant |
| E3 | 50 | F | Oral cavity | Squamous cell carcinoma | 1 | T2N0M0 |  | Doc040310 | malignant |
| E4 | 50 | F | Oral cavity | Squamous cell carcinoma | 1 | T2N0M0 |  | Doc040310 | malignant |
| E5 | 50 | F | Oral cavity | Squamous cell carcinoma | 1 | T2N0M0 |  | Doc040310 | malignant |
| E6 | 50 | F | Oral cavity | Squamous cell carcinoma | 1 | T1N0M0 |  | Doc040432 | malignant |
| E7 | 50 | F | Oral cavity | Squamous cell carcinoma | 1 | T1N0M0 |  | Doc040432 | malignant |
| E8 | 50 | F | Oral cavity | Squamous cell carcinoma | 1 | T1N0M0 |  | Doc040432 | malignant |
| E9 | 60 | M | Oral cavity | Squamous cell carcinoma | 1 | T2N0M0 |  | Doc060270 | malignant |
| E10 | 60 | M | Oral cavity | Squamous cell carcinoma | 1 | T2N0M0 |  | Doc060270 | malignant |
| E11 | 60 | M | Oral cavity | Squamous cell carcinoma | 1 | T2N0M0 |  | Doc060270 | malignant |
| E12 | 51 | M | Oral cavity | Squamous cell carcinoma | 1 | T4N0M0 |  | Doc060773 | malignant |
| E13 | 51 | M | Oral cavity | Squamous cell carcinoma | 1 | T4N0M0 |  | Doc060773 | malignant |
| E14 | 51 | M | Oral cavity | Squamous cell carcinoma | 1 | T4N0M0 |  | Doc060773 | malignant |
| E15 | 57 | F | Oral cavity | Squamous cell carcinoma | 1 | T4N0M0 |  | Doc060703 | malignant |
| E16 | 57 | F | Oral cavity | Squamous cell carcinoma | 1 | T4N0M0 |  | Doc060703 | malignant |
| F1 | 57 | F | Oral cavity | Squamous cell carcinoma | 1 | T4N0M0 |  | Doc060703 | malignant |
| F2 | 76 | M | Oral cavity | Squamous cell carcinoma | 1 | T1N0M0 |  | Doc031829 | malignant |
| F3 | 76 | M | Oral cavity | Squamous cell carcinoma | 1 | T1N0M0 |  | Doc031829 | malignant |
| F4 | 76 | M | Oral cavity | Squamous cell carcinoma | 1 | T1N0M0 |  | Doc031829 | malignant |
| F5 | 63 | M | Oral cavity | Squamous cell carcinoma | 1 | T1N0M0 |  | Doc060627 | malignant |
| F6 | 63 | M | Oral cavity | Squamous cell carcinoma | 1 | T1N0M0 |  | Doc060627 | malignant |
| F7 | 63 | M | Oral cavity | Squamous cell carcinoma | 1 | T1N0M0 |  | Doc060627 | malignant |
| F8 | 59 | M | Oral cavity | Squamous cell carcinoma | 1 | T2N0M0 |  | Doc040421 | malignant |
| F9 | 59 | M | Oral cavity | Squamous cell carcinoma | 1 | T2N0M0 |  | Doc040421 | malignant |
| F10 | 59 | M | Oral cavity | Squamous cell carcinoma | 1 | T2N0M0 |  | Doc040421 | malignant |
| F11 | 55 | M | Oral cavity | Squamous cell carcinoma | 1 | T1N0M0 |  | Doc040382 | malignant |
| F12 | 55 | M | Oral cavity | Squamous cell carcinoma | 1 | T1N0M0 |  | Doc040382 | malignant |
| F13 | 55 | M | Oral cavity | Squamous cell carcinoma | 1 | T1N0M0 |  | Doc040382 | malignant |
| F14 | 82 | F | Oral cavity | Squamous cell carcinoma | 1 | T1N0M0 |  | Doc040185 | malignant |
| F15 | 82 | F | Oral cavity | Squamous cell carcinoma | 1 | T1N0M0 |  | Doc040185 | malignant |
| F16 | 82 | F | Oral cavity | Squamous cell carcinoma | 1 | T1N0M0 |  | Doc040185 | malignant |
| G1 | 67 | F | Oral cavity | Squamous cell carcinoma | 1 -- 2 | T3N0M0 |  | Doc060099 | malignant |
| G2 | 67 | F | Oral cavity | Squamous cell carcinoma | 1 -- 2 | T3N0M0 |  | Doc060099 | malignant |
| G3 | 67 | F | Oral cavity | Squamous cell carcinoma | 1 -- 2 | T3N0M0 |  | Doc060099 | malignant |
| G4 | 47 | F | Oral cavity | Squamous cell carcinoma | 1 | T2N0M0 |  | Doc060073 | malignant |
| G5 | 47 | F | Oral cavity | Squamous cell carcinoma | 1 | T2N0M0 |  | Doc060073 | malignant |
|  |  |  |  |  |  |  |  |  |  |

| G6 | 47 | F | Oral cavity | Squamous cell carcinoma | 1 | T2N0M0 |  | Doc060073 | malignant |
| --- | --- | --- | --- | --- | --- | --- | --- | --- | --- |
| G7 | 61 | M | Oral cavity | Squamous cell carcinoma | 1 | T1N0M0 |  | Doc040354 | malignant |
| G8 | 61 | M | Oral cavity | Squamous cell carcinoma | 1 | T1N0M0 |  | Doc040354 | malignant |
| G9 | 61 | M | Oral cavity | Squamous cell carcinoma | 1 | T1N0M0 |  | Doc040354 | malignant |
| G10 | 72 | M | Oral cavity | Squamous cell carcinoma | 1 | T1N0M0 |  | Doc060012 | malignant |
| G11 | 72 | M | Oral cavity | Squamous cell carcinoma | 1 | T1N0M0 |  | Doc060012 | malignant |
| G12 | 72 | M | Oral cavity | Squamous cell carcinoma | 1 | T1N0M0 |  | Doc060012 | malignant |
| G13 | 62 | F | Oral cavity | Squamous cell carcinoma | 2 | T1N0M0 |  | Doc051693 | malignant |
| G14 | 62 | F | Oral cavity | Squamous cell carcinoma | 2 | T1N0M0 |  | Doc051693 | malignant |
| G15 | 62 | F | Oral cavity | Squamous cell carcinoma | 2 | T1N0M0 |  | Doc051693 | malignant |
| G16 | 51 | M | Oral cavity | Squamous cell carcinoma | 1 | T1N0M0 |  | Doc051131 | malignant |
| H1 | 51 | M | Oral cavity | Squamous cell carcinoma | 1 | T1N0M0 |  | Doc051131 | malignant |
| H2 | 51 | M | Oral cavity | Squamous cell carcinoma | 1 | T1N0M0 |  | Doc051131 | malignant |
| H3 | 66 | F | Oral cavity | Squamous cell carcinoma (hyperplasia of squamous epithelium) | - | T1N0M0 |  | Doc051124 | malignant |
| H4 | 66 | F | Oral cavity | Squamous cell carcinoma (hyperplasia of squamous epithelium) | - | T1N0M0 |  | Doc051124 | malignant |
| H5 | 66 | F | Oral cavity | Squamous cell carcinoma | 1 | T1N0M0 |  | Doc051124 | malignant |
| H6 | 55 | F | Oral cavity | Squamous cell carcinoma | 1 | T1N0M0 |  | Doc051010 | malignant |
| H7 | 55 | F | Oral cavity | Squamous cell carcinoma | 1 | T1N0M0 |  | Doc051010 | malignant |
| H8 | 55 | F | Oral cavity | Squamous cell carcinoma | 1 | T1N0M0 |  | Doc051010 | malignant |
| H9 | 73 | M | Oral cavity | Squamous cell carcinoma | 1 | T2N0M0 |  | Doc062228 | malignant |
| H10 | 73 | M | Oral cavity | Squamous cell carcinoma | 1 | T2N0M0 |  | Doc062228 | malignant |
| H11 | 73 | M | Oral cavity | Squamous cell carcinoma | 1 | T2N0M0 |  | Doc062228 | malignant |
| H12 | 61 | M | Oral cavity | Squamous cell carcinoma | 1 | T1N0M0 |  | Doc062247 | malignant |
| H13 | 61 | M | Oral cavity | Squamous cell carcinoma | 1 | T1N0M0 |  | Doc062247 | malignant |
| H14 | 61 | M | Oral cavity | Squamous cell carcinoma (necrotic and hyalinosis tissue) | - | T1N0M0 |  | Doc062247 | malignant |
| H15 | 75 | M | Oral cavity | Squamous cell carcinoma | 1 | T2N0M0 |  | Doc062210 | malignant |
| H16 | 75 | M | Oral cavity | Squamous cell carcinoma | 1 | T2N0M0 |  | Doc062210 | malignant |
| I1 | 75 | M | Oral cavity | Squamous cell carcinoma | 1 | T2N0M0 |  | Doc062210 | malignant |
| I2 | 40 | F | Oral cavity | Squamous cell carcinoma | 1 | T2N0M0 |  | Doc062195 | malignant |
| I3 | 40 | F | Oral cavity | Squamous cell carcinoma | 1 | T2N0M0 |  | Doc062195 | malignant |
| I4 | 40 | F | Oral cavity | Squamous cell carcinoma | 1 | T2N0M0 |  | Doc062195 | malignant |
| I5 | 55 | M | Oral cavity | Squamous cell carcinoma | 1 | T3N0M0 |  | Doc061830 | malignant |
| I6 | 55 | M | Oral cavity | Squamous cell carcinoma | 1 | T3N0M0 |  | Doc061830 | malignant |
| I7 | 55 | M | Oral cavity | Squamous cell carcinoma | 1 | T3N0M0 |  | Doc061830 | malignant |
| I8 | 70 | F | Oral cavity | Squamous cell carcinoma | 1 | T3N0M0 |  | Doc061227 | malignant |
| I9 | 70 | F | Oral cavity | Squamous cell carcinoma | 1 | T3N0M0 |  | Doc061227 | malignant |
| I10 | 70 | F | Oral cavity | Squamous cell carcinoma | 1 | T3N0M0 |  | Doc061227 | malignant |
| I11 | 56 | M | Oral cavity | Squamous cell carcinoma | 1 | T2N0M0 |  | Doc061174 | malignant |
|  |  |  |  |  |  |  |  |  |  |

| I12 | 56 | M | Oral cavity | Squamous cell carcinoma | 1 | T2N0M0 |  | Doc061174 | malignant |
| --- | --- | --- | --- | --- | --- | --- | --- | --- | --- |
| I13 | 56 | M | Oral cavity | Squamous cell carcinoma | 1 | T2N0M0 |  | Doc061174 | malignant |
| I14 | 51 | F | Oral cavity | Squamous cell carcinoma | 1 | T3N1M1 |  | Doc061134 | malignant |
| I15 | 51 | F | Oral cavity | Squamous cell carcinoma (fibrous tissue) | - | T3N1M1 |  | Doc061134 | malignant |
| I16 | 51 | F | Oral cavity | Squamous cell carcinoma (sparse) | 1 | T3N1M1 |  | Doc061134 | malignant |
| J1 | 38 | M | Oral cavity | Squamous cell carcinoma | 1 | T1N0M0 |  | Doc031980 | malignant |
| J2 | 38 | M | Oral cavity | Squamous cell carcinoma | 1 | T1N0M0 |  | Doc031980 | malignant |
| J3 | 38 | M | Oral cavity | Squamous cell carcinoma | 1 | T1N0M0 |  | Doc031980 | malignant |
| J4 | 45 | M | Oral cavity | Squamous cell carcinoma | 1 | T2N0M0 |  | Doc040434 | malignant |
| J5 | 45 | M | Oral cavity | Squamous cell carcinoma | 1 | T2N0M0 |  | Doc040434 | malignant |
| J6 | 45 | M | Oral cavity | Squamous cell carcinoma | 1 | T2N0M0 |  | Doc040434 | malignant |
| J7 | 52 | F | Oral cavity | Squamous cell carcinoma (sparse) | - | T2N0M0 |  | Doc060946 | malignant |
| J8 | 52 | F | Oral cavity | Squamous cell carcinoma | 2 | T2N0M0 |  | Doc060946 | malignant |
| J9 | 52 | F | Oral cavity | Squamous cell carcinoma | 2 | T2N0M0 |  | Doc060946 | malignant |
| J10 | 64 | M | Oral cavity | Squamous cell carcinoma | 2 | T1N0M0 |  | Doc050208 | malignant |
| J11 | 64 | M | Oral cavity | Squamous cell carcinoma | 2 | T1N0M0 |  | Doc050208 | malignant |
| J12 | 64 | M | Oral cavity | Squamous cell carcinoma | 2 | T1N0M0 |  | Doc050208 | malignant |
| J13 | 55 | M | Oral cavity | Squamous cell carcinoma | 2 | T1N0M0 |  | Doc062087 | malignant |
| J14 | 55 | M | Oral cavity | Squamous cell carcinoma | 2 | T1N0M0 |  | Doc062087 | malignant |
| J15 | 55 | M | Oral cavity | Squamous cell carcinoma | 2 | T1N0M0 |  | Doc062087 | malignant |
| J16 | 50 | M | Oral cavity | Squamous cell carcinoma | 2 | T3N0M0 |  | Doc060756 | malignant |
| K1 | 50 | M | Oral cavity | Squamous cell carcinoma | 2 | T3N0M0 |  | Doc060756 | malignant |
| K2 | 50 | M | Oral cavity | Squamous cell carcinoma | 2 | T3N0M0 |  | Doc060756 | malignant |
| K3 | 49 | M | Oral cavity | Squamous cell carcinoma (skeletal muscle tissue) | - | T2N0M0 |  | Doc062436 | malignant |
| K4 | 49 | M | Oral cavity | Squamous cell carcinoma (skeletal muscle tissue) | - | T2N0M0 |  | Doc062436 | malignant |
| K5 | 49 | M | Oral cavity | Squamous cell carcinoma (sparse) | - | T2N0M0 |  | Doc062436 | malignant |
| K6 | 42 | M | Oral cavity | Squamous cell carcinoma | 2 | T4N0M0 |  | Doc060462 | malignant |
| K7 | 42 | M | Oral cavity | Squamous cell carcinoma | 2 | T4N0M0 |  | Doc060462 | malignant |
| K8 | 42 | M | Oral cavity | Squamous cell carcinoma (sparse) | 2 | T4N0M0 |  | Doc060462 | malignant |
| K9 | 50 | M | Oral cavity | Squamous cell carcinoma | 1 -- 2 | TxN0M0 |  | Doc041188 | malignant |
| K10 | 50 | M | Oral cavity | Squamous cell carcinoma | 1 -- 2 | TxN0M0 |  | Doc041188 | malignant |
| K11 | 50 | M | Oral cavity | Squamous cell carcinoma | 1 -- 2 | TxN0M0 |  | Doc041188 | malignant |
| K12 | 43 | M | Oral cavity | Squamous cell carcinoma | 2 | T2N0M0 |  | Doc041473 | malignant |
| K13 | 43 | M | Oral cavity | Squamous cell carcinoma | 2 | T2N0M0 |  | Doc041473 | malignant |
| K14 | 43 | M | Oral cavity | Squamous cell carcinoma | 2 | T2N0M0 |  | Doc041473 | malignant |
| K15 | 50 | M | Oral cavity | Squamous cell carcinoma | 3 | TxN0M0 |  | Doc060153 | malignant |
| K16 | 50 | M | Oral cavity | Squamous cell carcinoma | 3 | TxN0M0 |  | Doc060153 | malignant |
| L1 | 50 | M | Oral cavity | Squamous cell carcinoma | 3 | TxN0M0 |  | Doc060153 | malignant |
|  |  |  |  |  |  |  |  |  |  |

| L2 | 56 | F | Oral cavity | Squamous cell carcinoma | 3 | T4N0M0 |  | Doc040532 | malignant |
| --- | --- | --- | --- | --- | --- | --- | --- | --- | --- |
| L3 | 56 | F | Oral cavity | Squamous cell carcinoma | 3 | T4N0M0 |  | Doc040532 | malignant |
| L4 | 56 | F | Oral cavity | Squamous cell carcinoma | 3 | T4N0M0 |  | Doc040532 | malignant |
| L5 | 50 | M | Oral cavity | Normal lingual tissue | - | - |  | Doc06N011 | normal |
| L6 | 50 | M | Oral cavity | Normal lingual tissue (skeletal muscle and adipose tissue) | - | - |  | Doc06N011 | normal |
| L7 | 50 | M | Oral cavity | Normal lingual tissue (glandular organ tissue) | - | - |  | Doc06N011 | normal |
| L8 | 46 | M | Oral cavity | Normal lingual tissue | - | - |  | Doc06N018 | normal |
| L9 | 46 | M | Oral cavity | Normal lingual tissue | - | - |  | Doc06N018 | normal |
| L10 | 46 | M | Oral cavity | Normal lingual tissue | - | - |  | Doc06N018 | normal |
| L11 | 19 | M | Oral cavity | Normal lingual tissue (skeletal muscle tissue) | - | - |  | Doc06N027 | normal |
| L12 | 19 | M | Oral cavity | Normal lingual tissue | - | - |  | Doc06N027 | normal |
| L13 | 19 | M | Oral cavity | Normal lingual tissue (skeletal muscle tissue) | - | - |  | Doc06N027 | normal |
| L14 | 21. Day | M | Oral cavity | Normal lingual tissue | - | - |  | Doc06N025 | normal |
| L15 | 21. Day | M | Oral cavity | Normal lingual tissue | - | - |  | Doc06N025 | normal |
| L16 | 21. Day | M | Oral cavity | Normal lingual tissue | - | - |  | Doc06N025 | normal |
| M1 | 21 | F | Oral cavity | Normal lingual tissue | - | - |  | Doc06N024 | normal |
| M2 | 21 | F | Oral cavity | Normal lingual tissue | - | - |  | Doc06N024 | normal |
| M3 | 21 | F | Oral cavity | Normal lingual epithelium tissue (sparse) | - | - |  | Doc06N024 | normal |
| M4 | 21 | F | Oral cavity | Normal lingual epithelium tissue (sparse) | - | - |  | Doc06N023 | normal |
| M5 | 21 | F | Oral cavity | Normal lingual tissue | - | - |  | Doc06N023 | normal |
| M6 | 21 | F | Oral cavity | Normal lingual tissue | - | - |  | Doc06N023 | normal |
| M7 | 21 | F | Oral cavity | Normal lingual tissue | - | - |  | 06N23---41 | normal |
| M8 | 21 | F | Oral cavity | Normal lingual tissue | - | - |  | 06N23---41 | normal |
| M9 | 21 | F | Oral cavity | Normal lingual tissue | - | - |  | 06N23---41 | normal |
| M10 | 15 | F | Oral cavity | Normal lingual tissue | - | - |  | Doc06N002 | normal |
| M11 | 15 | F | Oral cavity | Normal lingual tissue | - | - |  | Doc06N002 | normal |
| M12 | 15 | F | Oral cavity | Normal lingual tissue | - | - |  | Doc06N002 | normal |
| M13 | 16 | M | Oral cavity | Normal lingual tissue | - | - |  | Doc06N007 | normal |
| M14 | 16 | M | Oral cavity | Normal lingual tissue | - | - |  | Doc06N007 | normal |
| M15 | 16 | M | Oral cavity | Normal lingual tissue | - | - |  | Doc06N007 | normal |
| M16 | 58 | M | Skin | Marked point | - | - |  | Kin070024 | normal |
