# Supplementary material for: Targeting CCL2-CCR4 axis suppress cell migration of head and neck squamous cell carcinoma
Source: Cell Death Dis. 2022 Feb 17;13(2):158. doi: 10.1038/s41419-022-04610-5 (PMC8854715; doi:10.1038/s41419-022-04610-5)
Supplement: Supplementary file 15 — Supplementary Table 3 [file 41419_2022_4610_MOESM15_ESM.docx]

**Supplementary Table 3. Sequences of each siRNA**

| **siRNA** | **NO.** | **Target Sequence** |
| --- | --- | --- |
| **CCR2** | **1** | **TGTATCACATCGGTTATTT** |
|  | 2 | CACATCTCGTTCTCGGTTT |
|  | 3 | TACCAACGAGAGCGGTGAA |
| **CCR4** | 1 | CTCGATGAAAGCATATACA |
|  | 2 | CCCACGGATATAGCAGACA |
|  | **3** | **GTTATACTGAGCGCAACCA** |
| **VAV2** | 1 | AGAGGGTGCTCAAATACCA |
|  | **2** | **GAAAGTCTGCCACGATAAA** |
|  | 3 | GCTTTGCAATAAGCATCAA |
| **MYL2** | 1 | GAGTGAACGTGAAAAATGA |
|  | 2 | GGGTCCAATTAACTTTACT |
|  | **3** | **CGGAGAGGTTTTCCAAGGA** |
