# Supplementary material for: Targeting CCL2-CCR4 axis suppress cell migration of head and neck squamous cell carcinoma
Source: Cell Death Dis. 2022 Feb 17;13(2):158. doi: 10.1038/s41419-022-04610-5 (PMC8854715; doi:10.1038/s41419-022-04610-5)
Supplement: Supplementary file 16 — Supplementary Table 4 [file 41419_2022_4610_MOESM16_ESM.docx]

**Supplementary Table 4. Sequences of each primers** **used for qRT-PCR**

| **Names** | **5’ to 3’** | **Sequence** |
| --- | --- | --- |
| CCL2 | Forward primer  Reverse primer | CTTGGGTTGTGGAGTGAGTGT  AGCAGAAGTGGGTTCAGGATT |
| CCR2 | Forward primer  Reverse primer | CTTCATCATCCTCCTGACAATCG  GGAAAATAAGGGCCACAGACATA |
| CCR4 | Forward primer  Reverse primer | CGCCTTGTTCTTCTTCTCATTTT  TGGCTTTCTGTTCAGCACTTG |
| CCL17 | Forward primer  Reverse primer | AGCCATTCCCCTTAGAAA  TCTTCACTCTCTTGTTGTTGG |
| CCL22 | Forward primer  Reverse primer | ATCTGGGTTCCATCTCTGTCTCC  AGAAGTGGGATGTGTAGTATGGGTT |
| VAV2 | Forward primer  Reverse primer | TGTGGTCCTACGGCTTCTAC  ACTGTGGTGGTTGGCATTG |
| VAV3 | Forward primer  Reverse primer | ATCGCTCGGTATGACTTCTGT  CTCCTCTCCACCAGCCATT |
| PREX1 | Forward primer  Reverse primer | GCTGCTGTCCACCATCAC  TCCTGTCCATCTCGCTCTC |
| PREX2 | Forward primer  Reverse primer | AGCAGGAGATGAACAGGAAGAT  GGTGAAGGAGGCGATGGA |
| ECT2 | Forward primer  Reverse primer | GCTCCACTCCAGTTCCTTCA  CGTTGTCCTTCCTCTTCCAATG |
| MYL2 | Forward primer  Reverse primer | CACGACCTCCTGTTTATTG  ATTTATCCACCTCCATCTTC |
| GAPDH | Forward primer  Reverse primer | AAGAAGGTGGTGAAGCAGG  GTCAAAGGTGGAGGAGTGG |
