# Supplementary material for: Targeting CCL2-CCR4 axis suppress cell migration of head and neck squamous cell carcinoma
Source: Cell Death Dis. 2022 Feb 17;13(2):158. doi: 10.1038/s41419-022-04610-5 (PMC8854715; doi:10.1038/s41419-022-04610-5)
Supplement: Supplementary file 17 — Supplementary figure legends [file 41419_2022_4610_MOESM17_ESM.docx]

**Supplementary figure legends**

**Supplementary figure 1. Knockdown efficiency of each siRNA in HNSCC cells.**

For each target gene, we designed 3 different siRNA sequences and selected the sequence with best inhibition efficiency for the following experiment.

A to D, the qRT-PCR assay revealed the knockdown efficiency of 3 different siRNA sequences in HNSCC cells determined in RNA level.

E to L, the Western blot assay revealed the knockdown efficiency of 3 different siRNA sequences in HNSCC cells determined in protein level.

**Supplementary figure 2. The expression levels of CCL17 and CCL22 in HNSCC cells show only slight fluctuations after induction with CCL2.**

A and B. The CCL17 (A) and CCL22 (B) expression levels of HNSCC cells did not alter after induction with CCL2. ELISA assays revealed that both CCL17 and CCL22, other main ligands of CCR4, in cultured supernatants of HNSCC cells did not alter the following induction with CCL2 at 24h (NS, no statistical significance).

C. qRT-PCR data revealed that the relative mRNA expression levels of CCL17 slightly increased after induction with CCL2 in HSC6 cell lines. But the relative mRNA expression levels of CCL22 did not change after induction with CCL2 in HNSCC cell lines (*P < 0.05; NS, no statistical significance).

**Supplementary figure 3. siCCR2 could not inhibit the activation of Rac1 induced by CCL2.**

The amount of GTP-bound Rac1 of HNSCC cells with siCCR2 was detected and compared with that of the NC group. All the above cells were treated with exogenous CCL2 (100 ng/mL). Results showed that inhibition with CCR2 could not abolish the up-regulation of GTP-bound Rac1 induced by CCL2 (NS, no statistical significance).

**Supplementary figure 4. CCL2-CCR4 signaling, not CCL2-CCR2 signaling, induced the up-regulation of p-MLC through activation of Rac1.**

The levels of p-MLC of HNSCC cells with siCCR2 or siCCR4 were detected and compared with that of the NC group after cultured with or without Rac1 inhibitor (100 μM). Results indicated that CCR4 (not CCR2) inhibition could significantly suppress the up-regulation of p-MLC in HNSCC cells induced by CCL2. Moreover, results confirmed the crucial role of Rac1 in the up-regulation of p-MLC induced by CCL2-CCR4 signaling. (*P < 0.05; **P < 0.01; NS, no statistical significance).

**Supplementary figure 5. The relative expression levels of several GEFs in HNSCC cells induced by CCL2.**

HNSCC cells were treated with or without CCL2 (100 ng/mL) for 2 hours. qRT-PCR data revealed that the relative expression levels of several GEFs after induction with CCL2. The result indicated that the mRNA levels of Vav2, Prex1, and ECT2 increased significantly after induction with CCL2.

**Supplementary figure 6. CCL2 did not enhance the formation of the Prex1-Rac1 or ECT2-Rac1 complex in HNSCC cells.**

HNSCC cell lysates were subjected to immunoprecipitation (IP) with Rac1 antibody, followed by Western Blot assay of the co-immunoprecipitation with Prex1 or ECT2 antibody. Results revealed that the formation of Prex1-Rac1 or ECT2-Rac1complex did not significantly alter after treatment with CCL2.

**Supplementary figure 7. CCL2 induced the formation of the Vav2-Rac1 complex in an Src kinase-independent manner.**

HNSCC cells were incubated in culture medium with or without the serine kinase inhibitor (PP2, 1 μM) for 24 hours. The cells were then treated with exogenous CCL2 (100 ng/mL) for 2 hours. HNSCC cell lysates were subjected to immunoprecipitation (IP) with p-Vav2 antibody, followed by Western Blot assay of the immunoprecipitates with the Rac1 antibody. It was revealed that serine kinase inhibitors could not abolish the enhancement of Vav2-Rac1 complex formation induced by CCL2.

**Supplementary figure 8. Supplementary results of HNSCC cell proliferation induced by CCL2.**

A. Both CCL2 and CCR4 monoclonal antibodies did not significantly alter the cell cycle of HNSCC cells. Cell cycle assay of HNSCC cells after CCL2 (100 ng/mL) with or without CCR4 monoclonal antibody (1 ug/mL) treatment for 48 hours by flow cytometry. Results revealed that both CCL2 and CCR4 monoclonal antibodies did not significantly alter the cell cycle of HNSCC cells compared with that of the control group (NS, no statistical significance).

B and C. Both CCL2 and CCR4 monoclonal antibodies did not significantly alter HNSCC cell proliferation. Cell proliferation assay of HNSCC after CCL2 (100 ng/mL) with or without CCR4 monoclonal antibody (1ug/ml) treatment for 48 hours by CCK8 assay (B). Results revealed that both CCL2 and CCR4 monoclonal antibodies did not significantly alter HNSCC cell proliferation compared with that of the control group. In addition, colony formation of HNSCC cells after CCL2 (100 ng/mL) with or without CCR4 monoclonal antibody (1 ug/mL) treatment for 14 days (C) (NS, no statistical significance).
